# Endothelial GSDMD underlies LPS-induced systemic vascular injury and lethality

**DOI:** 10.1172/jci.insight.182398

**Published:** 2025-02-10

**Authors:** Enyong Su, Xiaoyue Song, Lili Wei, Junqiang Xue, Xuelin Cheng, Shiyao Xie, Hong Jiang, Ming Liu

**Affiliations:** 1 Department of Cardiology, Zhongshan Hospital, Fudan University, Shanghai, China.; 2 Shanghai Institute of Cardiovascular Diseases, State Key Laboratory of Cardiovascular Diseases, NHC Key Laboratory of Ischemic Heart Diseases, Key Laboratory of Viral Heart Diseases, Chinese Academy of Medical Sciences, National Clinical Research Center for Interventional Medicine, Shanghai, China.; 3 Shanghai Engineering Research Center of AI Technology for Cardiopulmonary Diseases, Zhongshan Hospital, Fudan University, Shanghai, China.; 4 Department of Cardiology, Shanghai Xuhui Central Hospital, Zhongshan-Xuhui Hospital, Shanghai, China.; 5 Department of Health Management Center, Zhongshan Hospital, and; 6 Department of General Practice, Zhongshan Hospital, Fudan University, Shanghai, China.; 7 Department of Cardiology, the First Affiliated Hospital of Zhengzhou University, Zhengzhou, China.; 8 Innovative Center for New Drug Development of Immune Inflammatory Diseases, Ministry of Education, Fudan University, Shanghai, China.

**Keywords:** Immunology, Infectious disease, Bacterial infections, Endothelial cells, Pharmacology

## Abstract

Endothelial injury destroys endothelial barrier integrity, triggering organ dysfunction and ultimately resulting in sepsis-related death. Considerable attention has been focused on identifying effective targets for inhibiting damage to endothelial cells to treat endotoxemia-induced septic shock. Global gasdermin D (*Gsdmd*) deletion reportedly prevents death caused by endotoxemia. However, the role of endothelial GSDMD in endothelial injury and lethality in lipopolysaccharide-induced (LPS-induced) endotoxemia and the underlying regulatory mechanisms are unknown. Here, we show that LPS increases endothelial GSDMD level in aortas and lung microvessels. We demonstrated that endothelial *Gsdmd* deficiency, but not myeloid cell *Gsdmd* deletion, protects against endothelial injury and death in mice with endotoxemia or sepsis. In vivo experiments suggested that hepatocyte GSDMD mediated the release of high-mobility group box 1, which subsequently binds to the receptor for advanced glycation end products in endothelial cells to cause systemic vascular injury, ultimately resulting in acute lung injury and lethality in shock driven by endotoxemia or sepsis. Additionally, inhibiting endothelial GSDMD activation via a polypeptide inhibitor alleviated endothelial damage and improved survival in a mouse model of endotoxemia or sepsis. These data suggest that endothelial GSDMD is a viable pharmaceutical target for treating endotoxemia and endotoxemia-induced sepsis.

## Abstract 2

Inhibiting endothelial GSDMD-mediated pyroptosis, rather than decreasing the myeloid cell GSDMD pyroptosis-triggered cytokine storm, prevents vascular injury and death in endotoxemia and sepsis.

## Graphical abstract

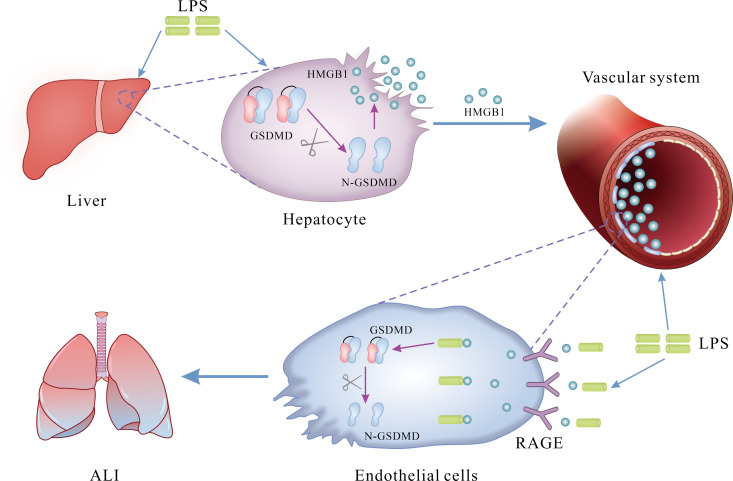

## Introduction

Sepsis is a life-threatening organ dysfunction caused by an aberrant host immune response to infection by pathogenic microorganisms (1). Approximately 48.9 million people worldwide are estimated to suffer from sepsis annually, approximately 22.5% of whom die, and the incidence and mortality of sepsis are still increasing (2, 3). Endothelial cells, as vascular barriers, are responsible for maintaining vascular homeostasis and the normal physiological function of multiple organs (4). During sepsis, vascular endothelial cell injury may lead to impaired microcirculation, tissue hypoperfusion, and organ failure (5, 6). To date, considerable attention has been focused on improving endothelial damage to treat sepsis (5, 7). However, few treatments targeting sepsis-induced endothelial injury have improved survival in large randomized clinical trials (5, 8).

Bacterial endotoxin (lipopolysaccharide [LPS]) is a main component of the outer membrane of Gram-negative bacteria and a potent immunostimulant (9). Gram-negative bacteria lyse and release large amounts of LPS into the circulation after infection, initiating a septicemic cascade (10, 11). Caspase-11 initiates the innate immune response once it senses LPS (12–14). Activated caspase-11 cleaves gasdermin D (GSDMD) into the N-terminal GSDMD fragment (GSDMD-N), which triggers the formation of pores in the plasma membrane, causing pyroptosis and the secretion of proinflammatory interleukin-1β (IL-1β) into the circulation (15–17).

High-mobility group box 1 (HMGB1), a DNA-binding protein, is abundantly expressed in the nucleus and regulates the immune response intracellularly and extracellularly upon infection (18–20). Both neutralizing HMGB1 and hepatocyte-specific *Hmgb1* deletion obviously reduce LPS-induced lethality (21–24). In vitro, LPS-induced HMGB1 release from hepatocytes requires the activation of the caspase-11/GSDMD signaling pathway, and HMGB1 subsequently delivers extracellular LPS into the cytosol of lung endothelial cells to induce endothelial pyroptosis (21).

The results of a recent study suggested that endothelial conditional *Caspase-11* deletion evidently reduced endotoxemia-induced lung microvascular injury and improved mouse survival from 0% to 50%–60% (25). Global knockout of *Gsdmd* protects against lethal endotoxemia caused by LPS challenge (26, 27). However, the role of endothelial GSDMD in LPS-induced endothelial injury and lethality and its regulatory mechanisms in vivo need to be further clarified. Here, we demonstrated that hepatocytic GSDMD-mediated release of HMGB1 bound with the receptor for advanced glycation end products (RAGE) and subsequently promoted vascular endothelial GSDMD activation and endothelial damage in mice induced by LPS or live bacteria, resulting in systemic vascular injury, acute lung injury (ALI), and death. Furthermore, conditional deletion or inhibition of endothelial *Gsdmd* protected mice from lethal endotoxic shock and sepsis.

## Results

### Endothelial Gsdmd deletion prevents endothelial damage-mediated vascular injury and death in endotoxemia.

**Figure 1:**
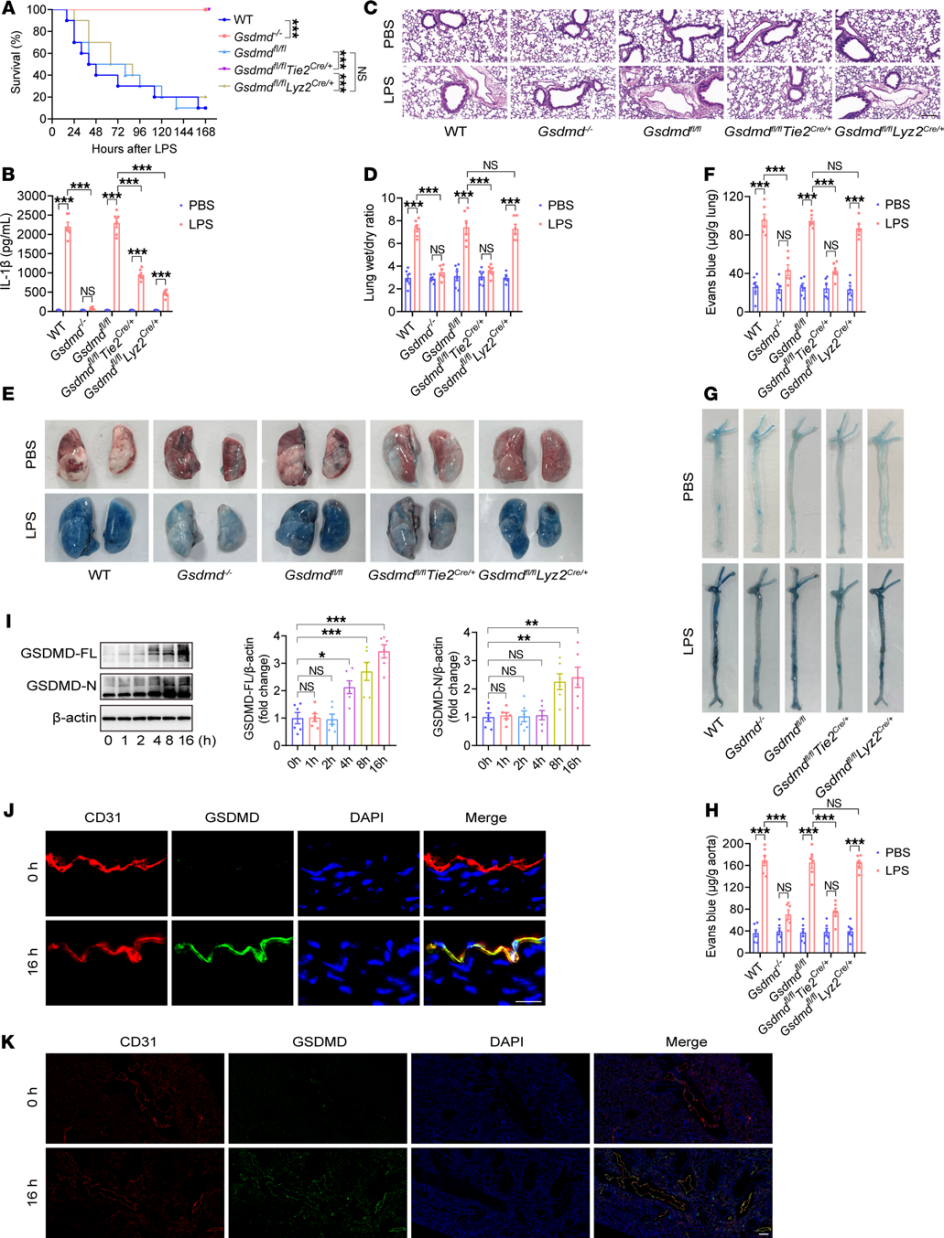
Endothelial *Gsdmd* deficiency protects against endothelial injury and death in endotoxemia. WT mice, *Gsdmd^–/–^* mice, *Gsdmd^fl/fl^* *Tie2^Cre/+^* mice, *Gsdmd^fl/fl^* *Lyz2^Cre/+^* mice, and *Gsdmd^fl/fl^* mice were intraperitoneally injected with a lethal dose of LPS (17.5 mg/kg). (**A**) The survival of the mice was monitored and is presented as a Kaplan-Meier plot. *n* = 10 per group. Survival data were compared via a log-rank (Mantel-Cox) test. Samples were obtained from WT mice, *Gsdmd^–/–^* mice, *Gsdmd^fl/fl^* *Tie2^Cre/+^* mice, *Gsdmd^fl/fl^* *Lyz2^Cre/+^* mice, and *Gsdmd^fl/fl^* mice after treatment with LPS (17.5 mg/kg) or PBS for 16 hours. (**B**) The plasma IL-1β concentration was determined. *n* = 6 per group. (**C**) Representative HE-stained images of the lung sections are presented. The scale bar represents 200 μm. (**D**) The lung wet/dry ratio was quantitatively analyzed. *n* = 6 per group. (**E**) Lung microvascular permeability was determined. (**F**) The lung Evans blue dye content was quantitatively analyzed. *n* = 6 per group. (**G**) Aortic permeability was determined. (**H**) The aortic Evans blue dye content was quantitatively analyzed. *n* = 6 per group. The data are expressed as the means ± SEMs. The data were analyzed by 2-way ANOVA with Bonferroni’s post hoc correction. WT mice were intraperitoneally injected with LPS (17.5 mg/kg) for different durations. (**I**) The protein expression levels of full-length GSDMD (GSDMD-FL) and GSDMD-N in whole aortas were evaluated by immunoblotting. *n* = 6 per group. The data are expressed as the means ± SEMs. The data were analyzed by 1-way ANOVA with Bonferroni’s post hoc correction. Representative immunofluorescence images of CD31 (red), GSDMD (green), and DAPI (blue) in (**J**) aortas and (**K**) lungs at 0 hour and 16 hours after exposure to LPS. The scale bar indicates 20 μm in the aorta and 200 μm in the lungs. All the data shown are representative of a minimum of 3 independent experiments. *P < 0.05, **P < 0.01, ***P < 0.001.

Endothelial cells and monocytes/macrophages primarily and actively participate in infection-initiated immune responses (28). GSDMD is required for LPS-induced pyroptosis and death (27, 29). To identify which specific GSDMD-expressing cells determine lethal endotoxemia, we constructed global *Gsdmd*-knockout (*Gsdmd^–/–^*) mice and crossed endothelial cell–specific *Cre* transgenic (*Tie2^Cre/+^*) mice or myeloid cell–specific *Cre* transgenic (*Lyz2^Cre/+^*) mice with *Gsdmd^fl/fl^* mice harboring *loxP*-flanked (floxed) alleles of *Gsdmd* to obtain endothelial *Gsdmd*-deficient (*Gsdmd^fl/fl^* *Tie2^Cre/+^*) mice, myeloid cell *Gsdmd*-deficient (*Gsdmd^fl/fl^* *Lyz2^Cre/+^*) mice, and their *Cre*-negative littermates (*Gsdmd^fl/fl^* mice) (Supplemental Figure 1, A and B; supplemental material available online with this article; https://doi.org/10.1172/jci.insight.182398DS1). We then intraperitoneally injected wild-type (WT) mice, *Gsdmd^–/–^* mice, *Gsdmd^fl/fl^* *Tie2^Cre/+^* mice, *Gsdmd^fl/fl^* *Lyz2^Cre/+^* mice, and *Gsdmd^fl/fl^* mice with LPS and observed its impact on survival for at least 1 week. Compared with that of *Gsdmd^fl/fl^* mice, the percentage survival of *Gsdmd^fl/fl^* *Tie2^Cre/+^* mice improved from 10% to 100%, which was comparable to that of *Gsdmd^–/–^* mice (Figure 1A). However, no difference in survival was observed between *Gsdmd^fl/fl^* mice and *Gsdmd^fl/fl^* *Lyz2^Cre/+^* mice (Figure 1A). Considering the gradual decrease in the survival rate of the mice 16 hours after the intraperitoneal injection of LPS, we used this key time point to observe the pathophysiological changes in endotoxemic mice. Circulating levels of IL-1β, which is released during pyroptotic cell death (9), were determined. No difference in the circulating IL-1β level was observed between *Gsdmd^–/–^* mice before and after LPS injection (Figure 1B). Compared with that in *Gsdmd^fl/fl^* mice, the plasma IL-1β concentration was obviously lower in *Gsdmd^fl/fl^* *Tie2^Cre/+^* mice and *Gsdmd^fl/fl^* *Lyz2^Cre/+^* mice following exposure to LPS but was significantly greater than the respective baseline level (Figure 1B). Compared with those in the PBS-injected mice, lung edema and lung microvascular permeability were significantly aggravated in the WT mice, *Gsdmd^fl/fl^* mice, and *Gsdmd^fl/fl^* *Lyz2^Cre/+^* mice after treatment with LPS but were prevented in the *Gsdmd^–/–^* mice and *Gsdmd^fl/fl^* *Tie2^Cre/+^* mice (Figure 1, C–F). As a whole organ and the largest artery, the aorta is a major vessel that delivers oxygenated blood from the left ventricle to multiple systemic organs (30). We investigated aortic changes in endotoxemic mice. Compared with PBS, LPS clearly increased aortic permeability in WT mice, which was consistent with the findings in *Gsdmd^fl/fl^* mice and *Gsdmd^fl/fl^* *Lyz2^Cre/+^* mice (Figure 1, G and H). Both global and endothelial *Gsdmd* deficiency significantly inhibited aortic permeability, indicating the integrity of the endothelial barrier (Figure 1, G and H). A significant increase in the expression and activation of GSDMD, which is expressed mainly in vascular endothelial cells, was observed in WT mice after 16 hours of treatment with LPS (Figure 1, I–K). However, LPS failed to change the endothelial GSDMD level in *Gsdmd^–/–^* mice (Supplemental Figure 1C). The level of endothelial GSDMD was elevated in *Gsdmd^fl/fl^* mice and *Gsdmd^fl/fl^* *Lyz2^Cre/+^* mice 16 hours after LPS injection but not in *Gsdmd^fl/fl^* *Tie2^Cre/+^* mice (Supplemental Figure 1D). These results suggest that endothelial *Gsdmd* deletion protects LPS-treated mice against systemic vascular injury and lethality.

### Hepatocyte Hmgb1 deficiency prevents vascular injury and death in endotoxemia.

HMGB1, which binds to LPS, regulates endothelial pyroptosis, causing endothelial injury in vitro (21). Circulating HMGB1 is derived mainly from hepatocytes during endotoxemia (21). To identify the effects of hepatocytic HMGB1 on the vascular system in vivo, we crossed hepatocyte-specific *Cre* transgenic (*Alb^Cre/+^*) mice with *Hmgb1^fl/fl^* mice harboring floxed alleles of *Hmgb1* to generate hepatocellular *Hmgb1*-deficient (*Hmgb1^fl/fl^* *Alb^Cre/+^*) mice and their *Cre*-negative littermates (*Hmgb1^fl/fl^* mice) (Supplemental Figure 2A). The survival of *Hmgb1^fl/fl^* *Alb^Cre/+^* mice was significantly improved compared with that of *Hmgb1^fl/fl^* mice with endotoxemia (Supplemental Figure 2B). No significant differences in the circulating HMGB1 or IL-1β concentrations were detected among *Hmgb1^fl/fl^* *Alb^Cre/+^* mice that were intraperitoneally administered LPS or PBS (Supplemental Figure 2, C and D). Compared with those in *Hmgb1^fl/fl^* mice, the LPS-induced plasma HMGB1 and IL-1β levels were significantly lower in *Hmgb1^fl/fl^* *Alb^Cre/+^* mice (Supplemental Figure 2, C and D). The conditional deletion of *Hmgb1* in hepatocytes clearly alleviated pulmonary edema, pulmonary microvascular permeability, and aortic permeability in endotoxemic mice (Supplemental Figure 2, E–H). Thus, hepatocyte-derived HMGB1 causes systemic vascular injury and death in endotoxemia.

### HMGB1 interacts with RAGE and subsequently participates in endothelial GSDMD-mediated vascular injury in endotoxemia.

**Figure 2:**
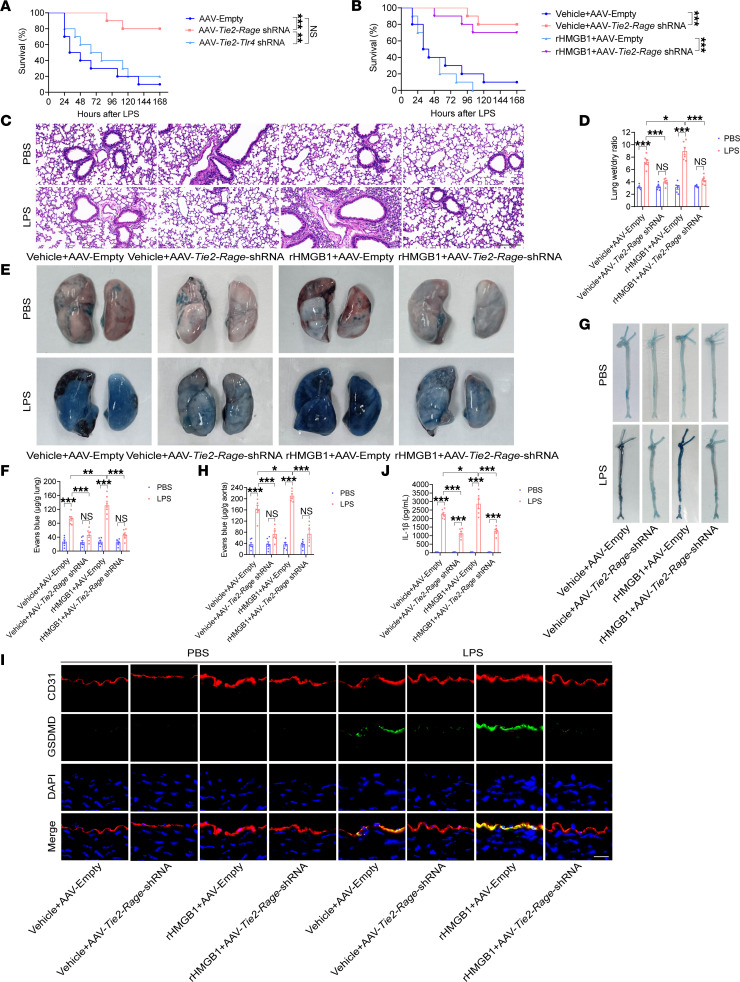
The HMGB1/RAGE signaling pathway increases endothelial GSDMD levels and promotes vascular injury in endotoxemia. Five-week-old WT mice were injected with an AAV9 vector via the tail vein. After 6 weeks, these mice were intraperitoneally injected with LPS (17.5 mg/kg). (**A**) A Kaplan-Meier survival plot of mice on the indicated days is presented. *n* = 10 per group. Survival data were compared via a log-rank (Mantel-Cox) test. Five-week-old WT mice were injected with an AAV9 vector. After 6 weeks, these mice were intraperitoneally injected with LPS (17.5 mg/kg). Subsequently, 5 μg rHMGB1 protein or vehicle was administered intravenously at 2, 16, 28, and 40 hours. (**B**) A Kaplan-Meier survival plot of mice is presented. *n* = 10 per group. Survival data were compared via a log-rank (Mantel-Cox) test. Five-week-old WT mice were injected with an AAV9 vector or an endothelial conditional *Rage* shRNA-knockdown AAV9 vector and were intraperitoneally injected with LPS (17.5 mg/kg) or PBS after 6 weeks. Then, 5 μg rHMGB1 protein or vehicle was administered intravenously at 2 and 16 hours. (**C**) HE staining of the lung sections is presented. The scale bar represents 200 μm. (**D**) The ratio of the wet lung weight to the dry lung weight was determined. *n* = 6 per group. (**E**) Lung microvascular permeability was assessed and (**F**) quantitatively analyzed. *n* = 6 per group. (**G**) Aortic permeability was assessed and (**H**) quantitatively analyzed. *n* = 6 per group. (**I**) Coimmunofluorescence staining of CD31 (red), GSDMD (green), and DAPI (blue) in aortas. The scale bar represents 20 μm. (**J**) The plasma IL-1β concentration was determined. *n* = 6 per group. The data are expressed as the means ± SEMs. The data were analyzed by 2-way ANOVA with Bonferroni’s post hoc correction. All the data shown are representative of a minimum of 3 independent experiments. *P < 0.05, **P < 0.01, ***P < 0.001.

RAGE and Toll-like receptor 4 (TLR4) act as pivotal HMGB1 receptors in the pathogenesis of inflammatory diseases (31, 32). We investigated the roles of these 2 transmembrane receptors in the endothelium during endotoxemia. Five-week-old WT mice were injected with a null adenoassociated virus serotype 9 (AAV9) vector or an endothelial conditional *Tlr4* or *Rage* shRNA-knockdown AAV9 vector via the tail vein, and these mice were treated with LPS 6 weeks later. Endothelial *Rage* knockdown significantly improved survival from 10% to 80% in LPS-treated mice (Figure 2A). No significant difference was detected between the mice injected with the null AAV9 vector and those injected with the endothelial cell–specific *Tlr4* shRNA-knockdown AAV9 vector (Figure 2A). Therefore, we further assessed the effects of the HMGB1/RAGE axis on endothelial GSDMD-induced vascular injury via in vivo experiments. Recombinant HMGB1 (rHMGB1) protein was administered intravenously at a dose of 5 μg at 2, 16, 28, and 40 hours after LPS injection. Compared with the vehicle, the rHMGB1 protein decreased the survival rate of the LPS-treated mice from 10% to 0%, which was significantly improved from 0% to 70% by the endothelial *Rage* shRNA-knockdown AAV9 vector (Figure 2B). Compared with the vehicle, the rHMGB1 protein significantly aggravated lung edema and increased lung microvascular permeability and aortic permeability, and these effects were reversed by inhibiting endothelial RAGE expression (Figure 2, C–H). Consistently, administration of the endothelial *Rage*-knockdown AAV9 vector significantly reduced the endothelial GSDMD level and the subsequent release of plasma IL-1β in LPS-treated mice stimulated with the rHMGB1 protein (Figure 2, I and J). To verify the necessity of the interaction of HMGB1 with RAGE in vascular injury, we also induced endotoxemia by intratracheally instilling LPS, which allowed the LPS to be evenly distributed in the lungs of the mice. Compared with the null AAV9 vector, the rHMGB1 protein–induced survival was significantly improved by the endothelial *Rage* shRNA-mediated knockdown of the AAV9 vector in LPS-treated mice (Supplemental Figure 3A). The effects of rHMGB1 on pulmonary edema, pulmonary microvascular permeability, aortic permeability, and the IL-1β concentration in LPS-induced mice were reversed by endothelial RAGE expression inhibition (Supplemental Figure 3, B–F). These data indicate that the interaction between HMGB1 and RAGE contributes to endothelial GSDMD-mediated systemic vascular injury and death in endotoxemia.

RAGE binds to various damage-associated molecular patterns, such as HMGB1 (33). FPS-ZM1, a RAGE inhibitor (34–36), was used to further elucidate whether the binding of HMGB1 to RAGE determines the vascular injury function of the HMGB1/RAGE axis in endotoxemia. WT mice were intraperitoneally injected with FPS-ZM1 (3 mg/kg) or dimethyl sulfoxide (DMSO) at 72, 48, 24, and 1 hour before LPS injection and at 24, 48, 72, 96, 120, 144, and 168 hours after LPS injection. rHMGB1 protein was administered intravenously at a dose of 5 μg at 2, 16, 28, and 40 hours after LPS injection. rHMGB1 protein–induced survival was significantly improved by FPS-ZM1 in LPS-treated mice (Supplemental Figure 4A). The effects of the rHMGB1 protein on lung edema, lung microvascular permeability, and aortic permeability in LPS-induced mice were reversed by FPS-ZM1 (Supplemental Figure 4, B–E). Consistently, the administration of FPS-ZM1 significantly reduced the release of plasma IL-1β in the LPS-treated mice stimulated with the rHMGB1 protein (Supplemental Figure 4F). Therefore, the binding of HMGB1 to RAGE is indispensable for systemic vascular injury in lethal endotoxic shock.

Alveolar epithelial cells provide protection against environmental insults, regulate water and ion transport, and produce pulmonary surfactants to maintain alveolar homeostasis (37). Injury to alveolar epithelial cells is an important factor in the occurrence and development of ALI (38). The absence of *Rage* protects mice from lethal endotoxemia and sepsis (21, 39). *Rage* mRNA is expressed in type II alveolar epithelial cells, and the protein expression level of RAGE in these cells steadily increases in response to LPS treatment (40, 41). Further exploration of the role of type II alveolar epithelial RAGE in endotoxemia is interesting. Five-week-old WT mice were injected with a null AAV9 vector, an endothelial conditional *Rage* shRNA-knockdown AAV9 vector, or a type II alveolar epithelial conditional *Rage* shRNA-knockdown AAV9 vector via the tail vein, and these mice were treated with LPS 6 weeks later. rHMGB1 protein was administered intravenously at a dose of 5 μg at 2, 16, 28, and 40 hours after LPS injection. Compared with the null AAV9 vector, the type II alveolar epithelial conditional *Rage* shRNA-mediated AAV9 vector increased survival from 0% to 30% in rHMGB1 protein–induced mice treated with LPS intraperitoneally or increased survival from 0% to 20% in rHMGB1 protein–induced mice treated intratracheally with LPS; however, no significant difference was observed between these groups (Supplemental Figure 5). Compared with the type II alveolar epithelial conditional *Rage* shRNA-knockdown AAV9 vector, the endothelial conditional *Rage* shRNA-knockdown AAV9 vector significantly improved survival in rHMGB1 protein–induced mice treated with LPS intraperitoneally or intratracheally (Supplemental Figure 5). Thus, endothelial RAGE rather than alveolar epithelial RAGE is essential for lethal endotoxemia.

### Hepatocyte Gsdmd deletion regulates HMGB1 release and inhibits vascular damage in endotoxemia.

**Figure 3:**
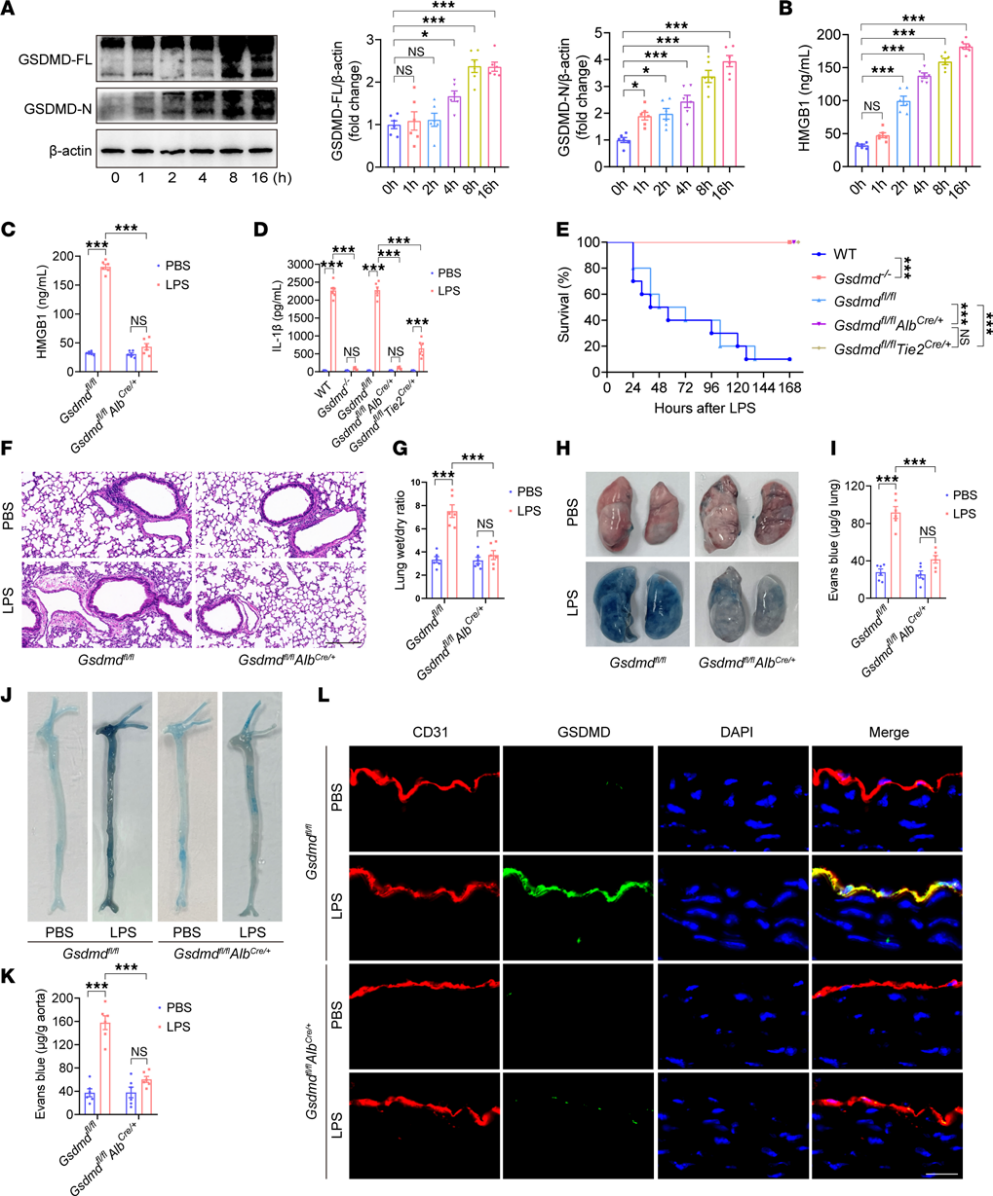
Deletion of hepatocyte *Gsdmd* decreases the endothelial GSDMD level and alleviates vascular injury in endotoxemia. WT mice were intraperitoneally injected with LPS (17.5 mg/kg) for different durations. (**A**) Representative immunoblots of GSDMD-FL and GSDMD-N protein expression in the liver. *n* = 6 per group. (**B**) The plasma HMGB1 concentration was measured. *n* = 6 per group. The data are expressed as the means ± SEMs. One-way ANOVA with Bonferroni’s post hoc correction was performed. WT, *Gsdmd^–/–^*, *Gsdmd^fl/fl^* *Tie2^Cre/+^*, *Gsdmd^fl/fl^* *Alb^Cre/+^*, and *Gsdmd^fl/fl^* mice were intraperitoneally injected with LPS (17.5 mg/kg) or PBS. After 16 hours, the plasma (**C**) HMGB1 and (**D**) IL-1β levels were determined. *n* = 6 per group. The data are shown as the means ± SEMs. Two-way ANOVA with Bonferroni’s post hoc correction was performed. (**E**) Mouse survival was assessed and is shown as a Kaplan-Meier plot. *n* = 10 per group. Survival data were analyzed by a log-rank (Mantel-Cox) test. Aortas and lungs were obtained from *Gsdmd^fl/fl^* *Alb^Cre/+^* and *Gsdmd^fl/fl^* mice after treatment with LPS (17.5 mg/kg) or PBS for 16 hours and were analyzed. (**F**) Representative HE-stained images of the lung sections. The scale bar represents 200 μm. (**G**) The lung wet/dry ratio was quantitatively analyzed. *n* = 6 per group. (**H**) Lung microvascular permeability was detected. (**I**) The lung Evans blue dye content was quantified. *n* = 6 per group. (**J**) Aortic permeability was determined. (**K**) The aortic Evans blue dye content was quantitatively analyzed. *n* = 6 per group. The data are shown as the means ± SEMs. Two-way ANOVA with Bonferroni’s post hoc correction was used. (**L**) Representative immunofluorescence images of CD31 (red), GSDMD (green), and DAPI (blue) staining in aortas. The scale bar represents 20 μm. All the data shown are representative of a minimum of 3 independent experiments. *P < 0.05, ***P < 0.001.

Hepatocyte-specific deletion of *Caspase-11* reduces the release of circulating HMGB1 and promotes survival from 0% to approximately 30% in LPS-treated mice (21). However, the effects of hepatocyte *Gsdmd* deletion on circulating HMGB1 levels and survival in endotoxin-treated mice are unknown. The GSDMD-N in the liver was significantly increased at 1 hour after treatment with LPS, which was earlier than the increase in plasma HMGB1 levels at 2 hours (Figure 3, A and B). We crossed *Alb^Cre/+^* mice with *Gsdmd^fl/fl^* mice to generate hepatocellular *Gsdmd*-deficient (*Gsdmd^fl/fl^* *Alb^Cre/+^*) mice and their *Cre*-negative littermates (*Gsdmd^fl/fl^* mice) (Supplemental Figure 6). Compared with those in *Gsdmd^fl/fl^* mice, the LPS-induced plasma HMGB1 and IL-1β levels were significantly lower in *Gsdmd^fl/fl^* *Alb^Cre/+^* mice, and no significant differences in the circulating HMGB1 and IL-1β concentrations were detected between *Gsdmd^fl/fl^* *Alb^Cre/+^* mice administered LPS or PBS (Figure 3, C and D). LPS-induced death in *Gsdmd^fl/fl^* *Alb^Cre/+^* mice was prevented, which was consistent with the findings in *Gsdmd^fl/fl^* *Tie2^Cre/+^* mice and *Gsdmd^–/–^* mice (Figure 3E). The conditional deletion of *Gsdmd* in hepatocytes clearly alleviated pulmonary edema and pulmonary microvascular permeability in endotoxemic mice (Figure 3, F–I). Compared with those in *Gsdmd^fl/fl^* mice, LPS-induced aortic permeability and endothelial GSDMD levels were significantly lower in *Gsdmd^fl/fl^* *Alb^Cre/+^* mice (Figure 3, J–L). These results indicate that hepatocyte GSDMD activation triggered HMGB1 release and caused endothelial GSDMD-mediated vascular injury and death in endotoxemia.

### Hepatocyte GSDMD regulates endothelial GSDMD-mediated vascular injury in an HMGB1-dependent manner in endotoxemia.

**Figure 4:**
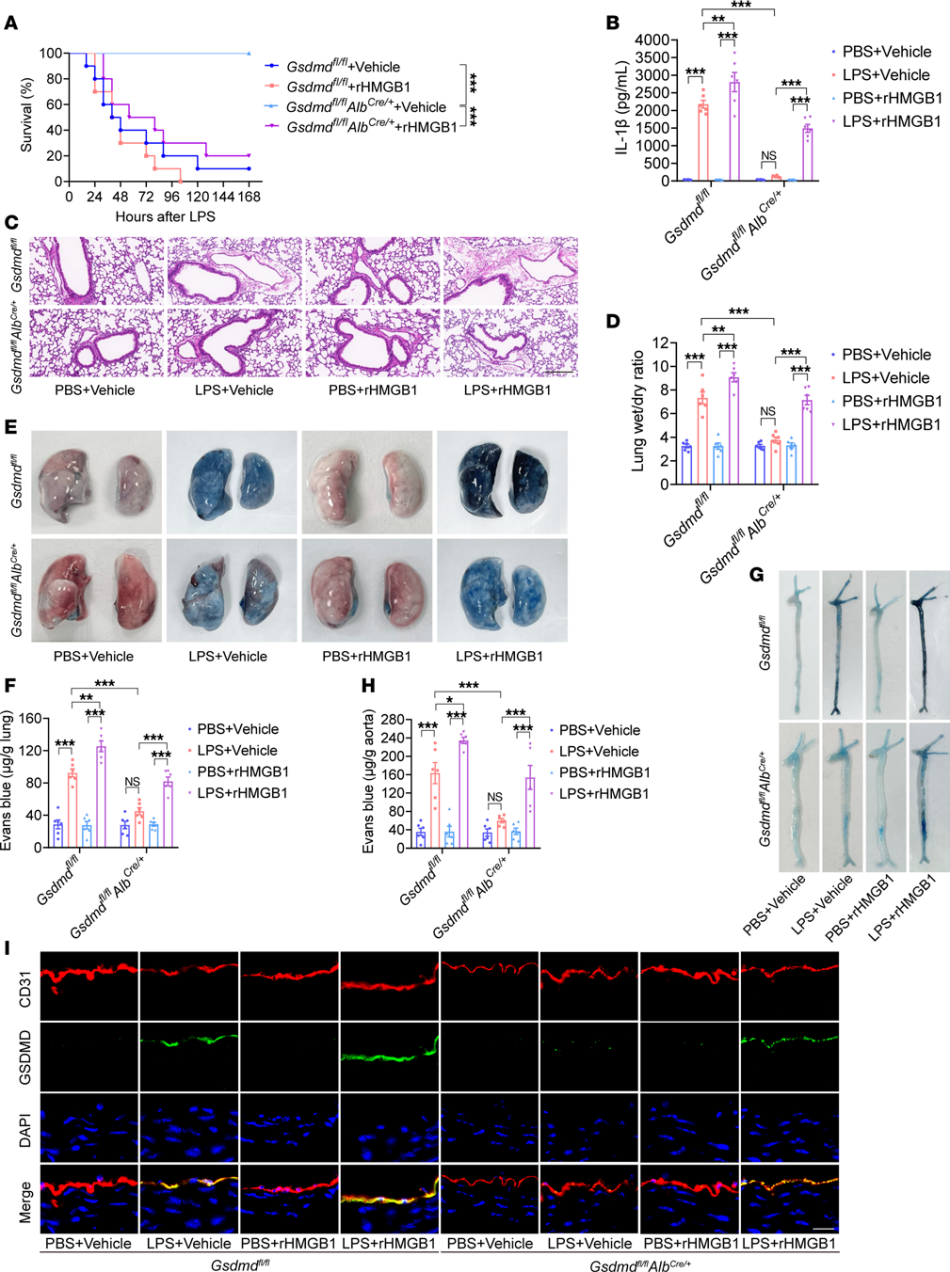
Hepatocyte GSDMD-mediated HMGB1 release regulates vascular injury and death in endotoxemia. *Gsdmd^fl/fl^Alb^Cre/+^* mice and *Gsdmd^fl/fl^* mice were intraperitoneally injected with LPS (17.5 mg/kg). The vehicle control or rHMGB1 protein was subsequently administered intravenously at a dose of 5 μg at 2, 16, 28, and 40 hours after LPS injection. (**A**) Mouse survival was assessed on the indicated days and is shown as a Kaplan-Meier plot. *n* = 10 per group. A log-rank (Mantel-Cox) test was used to compare survival curves. *Gsdmd^fl/fl^* *Alb^Cre/+^* mice and *Gsdmd^fl/fl^* mice were intraperitoneally injected with LPS (17.5 mg/kg) or PBS. Then, vehicle control or 5 μg of rHMGB1 protein was administered intravenously at 2 and 16 hours, and the blood, aortas, and lungs were excised from the mice and analyzed. (**B**) The plasma IL-1β level was determined. *n* = 6 per group. (**C**) H&E staining of the lung sections. The scale bar represents 200 μm. (**D**) The lung wet/dry ratio was quantitatively analyzed. *n* = 6 per group. (**E**) Lung microvascular permeability was determined by an Evans blue–albumin extravasation assay. (**F**) The contents of the extracted lung Evans blue dye were quantified. *n* = 6 per group. (**G**) Aortic permeability was determined by an Evans blue–albumin extravasation assay. (**H**) The aortic Evans blue dye content was quantitatively analyzed. *n* = 6 per group. The data are expressed as the means ± SEMs. The data were analyzed by 2-way ANOVA with Bonferroni’s post hoc correction. (**I**) Coimmunofluorescence staining of CD31 (red), GSDMD (green), and DAPI (blue) in aortas. The scale bar represents 20 μm. All the data shown are representative of a minimum of 3 independent experiments. *P < 0.05, **P < 0.01, ***P < 0.001.

The above data demonstrate that the plasma HMGB1 level is predominantly regulated by hepatocyte GSDMD activation in LPS-induced mice. In addition, HMGB1 delivers extracellular LPS into the cytosol to promote pulmonary endothelial pyroptosis in vitro (21). However, in vivo studies are needed to elucidate whether hepatocytic GSDMD regulates endothelial GSDMD-mediated vascular injury through the release of HMGB1 in endotoxemia. rHMGB1 protein can effectively increase plasma HMGB1 level in *Gsdmd^fl/fl^* *Alb^Cre/+^* mice treated with LPS (Supplemental Figure 7). Compared with vehicle administration, treatment with the rHMGB1 protein resulted in a decrease in survival and an evident increase in the IL-1β concentration in *Gsdmd^fl/fl^* *Alb^Cre/+^* mice treated with LPS (Figure 4, A and B). Compared with those in *Gsdmd^fl/fl^* mice, pulmonary edema, pulmonary microvascular permeability, and aortic permeability were obviously lower in *Gsdmd^fl/fl^* *Alb^Cre/+^* mice with endotoxemia, and these effects were reversed by the injection of the rHMGB1 protein (Figure 4, C–H). Compared with vehicle administration, rHMGB1 protein administration increased the endothelial GSDMD level in *Gsdmd^fl/fl^* *Alb^Cre/+^* mice treated with LPS (Figure 4I). Collectively, the results of the in vivo experiments suggest that endothelial GSDMD-mediated systemic vascular injury and lethality are dependent on hepatocytic GSDMD-mediated HMGB1 release in endotoxemia.

### Endothelial GSDMD contributes to vascular injury and death in sepsis.

**Figure 5:**
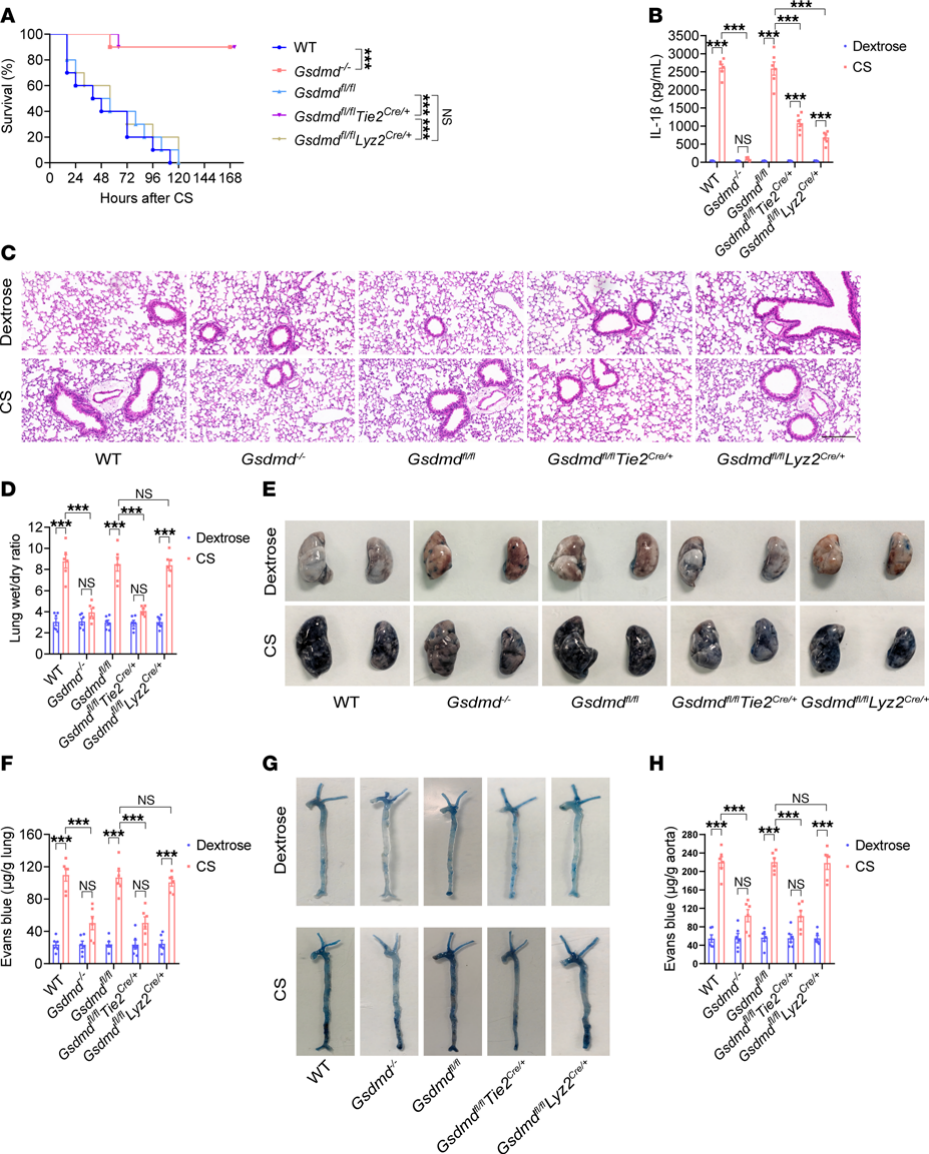
Endothelial *Gsdmd* deletion prevents vascular injury and death in sepsis. WT mice, *Gsdmd^–/–^* mice, *Gsdmd^fl/fl^* *Tie2^Cre/+^* mice, *Gsdmd^fl/fl^* *Lyz2^Cre/+^* mice, and *Gsdmd^fl/fl^* mice were intraperitoneally injected with a lethal dose of CS (2.5 mg/g). (**A**) The survival of the mice on the indicated days was monitored and is presented as a Kaplan-Meier plot. *n* = 10 per group. Survival data were compared via a log-rank (Mantel-Cox) test. Blood, aortas, and lungs were obtained from WT mice, *Gsdmd^–/–^* mice, *Gsdmd^fl/fl^* *Tie2^Cre/+^* mice, *Gsdmd^fl/fl^* *Lyz2^Cre/+^* mice, and *Gsdmd^fl/fl^* mice after treatment with CS (2.5 mg/g) or 5% dextrose for 16 hours. (**B**) The plasma IL-1β concentration was determined in the indicated groups. *n* = 6 per group. (**C**) Representative H&E-stained images of the lung sections are presented. The scale bar represents 200 μm. (**D**) The lung wet/dry ratio was quantitatively analyzed. *n* = 6 per group. (**E**) Lung microvascular permeability was determined by an Evans blue–albumin extravasation assay. (**F**) The amount of extracted pulmonary Evans blue dye in the formamide extracts was quantified by measuring the absorbance of the dye at 620 nm. *n* = 6 per group. (**G**) Aortic permeability was determined. (**H**) The aortic Evans blue dye content was quantitatively analyzed. *n* = 6 per group. The data are expressed as the means ± SEMs. The data were analyzed by 2-way ANOVA with Bonferroni’s post hoc correction. All the data shown are representative of a minimum of 3 independent experiments. ***P < 0.001.

We also assessed the role of endothelial GSDMD in sepsis. The cecal slurry (CS) model involves the intraperitoneal administration of the cecal contents of a euthanized animal into another animal and is used to induce polymicrobial sepsis (42, 43). We intraperitoneally injected CS into WT mice, *Gsdmd^–/–^* mice, *Gsdmd^fl/fl^* *Tie2^Cre/+^* mice, *Gsdmd^fl/fl^* *Lyz2^Cre/+^* mice, and *Gsdmd^fl/fl^* mice. Compared with that of *Gsdmd^fl/fl^* mice, the survival rate of *Gsdmd^fl/fl^* *Tie2^Cre/+^* mice improved from 0% to 90% in sepsis, which was comparable to that of *Gsdmd^–/–^* mice (Figure 5A). Because decreased survival of the mice was observed 16 hours after the intraperitoneal injection of CS, we performed biochemical and pathological tests at this time. No significant change in the circulating IL-1β level in *Gsdmd^–/–^* mice was observed after treatment with CS (Figure 5B). Compared with that in *Gsdmd^fl/fl^* mice, the plasma IL-1β concentration was obviously lower in *Gsdmd^fl/fl^* *Tie2^Cre/+^* mice and *Gsdmd^fl/fl^* *Lyz2^Cre/+^* mice following exposure to CS but significantly greater than the corresponding baseline level (Figure 5B). Compared with those of *Gsdmd^fl/fl^* mice, the lung edema, lung microvascular permeability, and aortic permeability of *Gsdmd^fl/fl^* *Tie2^Cre/+^* mice were significantly alleviated in sepsis (Figure 5, C–H). Survival, ALI, and systemic vascular injury were comparable between *Gsdmd^fl/fl^* mice and *Gsdmd^fl/fl^* *Lyz2^Cre/+^* mice with sepsis (Figure 5, A and C–H). These results suggest that endothelial *Gsdmd* deletion improved the integrity of the endothelial barrier, which protected mice against ALI and death in sepsis.

### The HMGB1/RAGE signaling pathway regulates vascular injury through endothelial GSDMD in sepsis.

To explore the regulatory mechanism of vascular injury in sepsis, WT mice were intraperitoneally injected with FPS-ZM1 (3 mg/kg) or DMSO at 72, 48, 24, and 1 hour before CS injection and at 24, 48, 72, 96, 120, 144, and 168 hours after CS injection. rHMGB1 protein was subsequently administered intravenously at a dose of 5 μg at 2, 16, 28, and 40 hours after CS injection. The survival of rHMGB1 protein–treated WT mice with sepsis significantly improved from 0% to 50% in response to FPS-ZM1 injection compared with that in response to DMSO injection (Supplemental Figure 8A). Compared with those of the DMSO-treated mice, the rHMGB1 protein–induced lung edema, lung microvascular permeability, aortic permeability, and plasma IL-1β levels of the WT mice with sepsis were obviously reversed by FPS-ZM1 (Supplemental Figure 8, B–F). These data indicate that the binding of HMGB1 to RAGE causes systemic vascular injury and death in sepsis.

**Figure 6:**
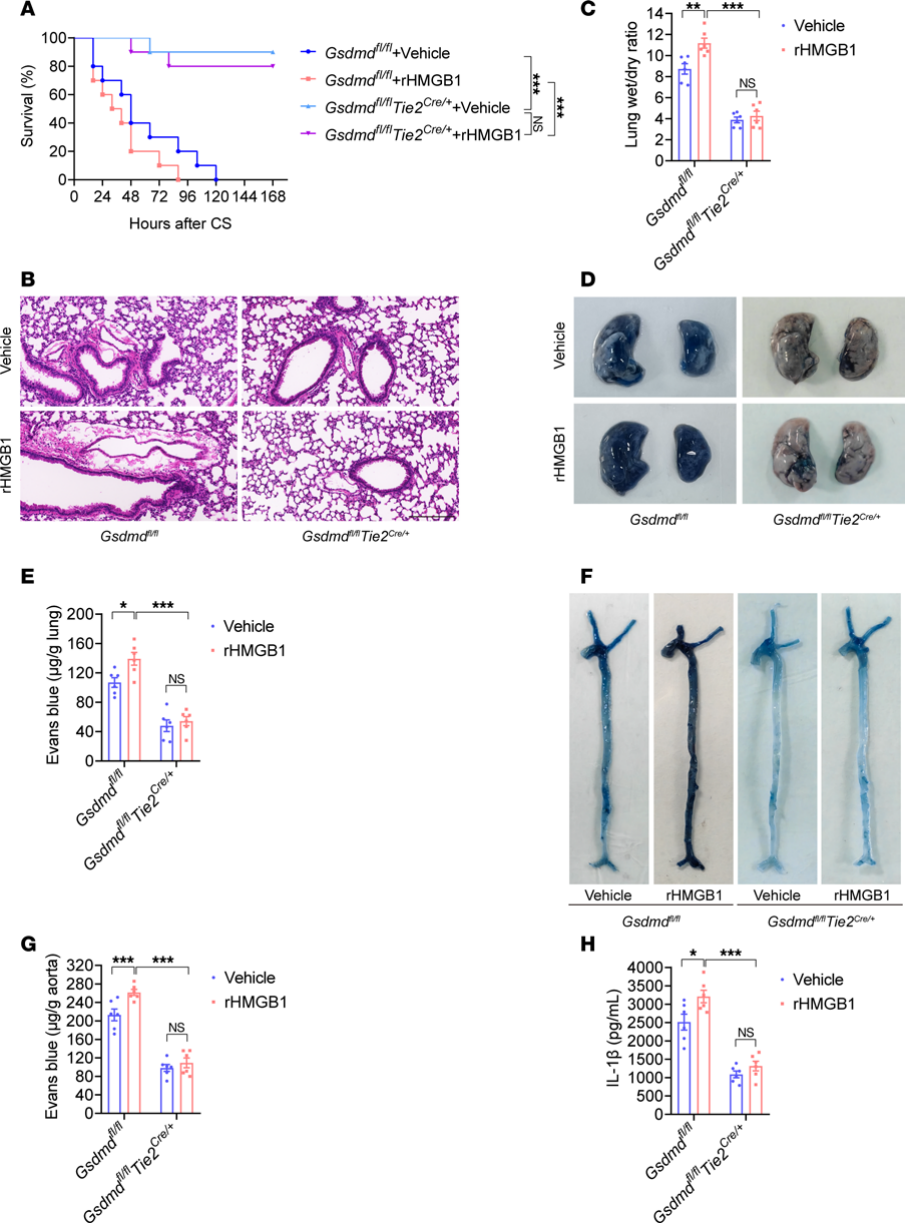
HMGB1 promotes vascular injury and death through endothelial GSDMD in sepsis. *Gsdmd^fl/fl^Tie2^Cre/+^* mice and *Gsdmd^fl/fl^* mice were intraperitoneally injected with CS (2.5 mg/g). The vehicle control or rHMGB1 protein was subsequently administered intravenously at a dose of 5 μg at 2, 16, 28, and 40 hours after CS injection. (**A**) Mouse survival was assessed on the indicated days and is shown as a Kaplan-Meier plot. *n* = 10 per group. A log-rank (Mantel-Cox) test was used to compare survival curves. *Gsdmd^fl/fl^* *Tie2^Cre/+^* mice and *Gsdmd^fl/fl^* mice were intraperitoneally injected with CS (2.5 mg/g). Then, vehicle control or 5 μg of rHMGB1 protein was administered intravenously at 2 and 16 hours, and the blood, aortas, and lungs were excised from the mice and analyzed. (**B**) H&E staining of the lung sections. The scale bar represents 200 μm. (**C**) The lung wet/dry ratio was quantitatively analyzed. *n* = 6 per group. (**D**) Lung microvascular permeability was determined by an Evans blue–albumin extravasation assay. (**E**) The contents of the extracted lung Evans blue dye were quantified. *n* = 6 per group. (**F**) Aortic permeability was determined by an Evans blue–albumin extravasation assay. (**G**) The aortic Evans blue dye content was quantitatively analyzed. *n* = 6 per group. (**H**) The plasma IL-1β level was determined. *n* = 6 per group. The data are expressed as the means ± SEMs. The data were analyzed by 2-way ANOVA with Bonferroni’s post hoc correction. All the data shown are representative of a minimum of 3 independent experiments. *P < 0.05, **P < 0.01, ***P < 0.001.

We further identified the underlying mechanism of the HMGB1/RAGE signaling pathway in vascular damage in sepsis. *Gsdmd^fl/fl^* *Tie2^Cre/+^* mice and *Gsdmd^fl/fl^* mice were administered CS and then treated intravenously with 5 μg of the rHMGB1 protein at 2, 16, 28, and 40 hours after CS injection. Compared with those in *Gsdmd^fl/fl^* mice, rHMGB1 protein–induced lethality, pulmonary edema, pulmonary microvascular permeability, aortic permeability, and IL-1β concentration were significantly lower in *Gsdmd^fl/fl^* *Tie2^Cre/+^* mice (Figure 6). Therefore, endothelial GSDMD mediates the regulatory effects of the HMGB1/RAGE axis on vascular injury and death in sepsis.

### Hepatocyte GSDMD regulates vascular injury through the release of HMGB1 in sepsis.

**Figure 7:**
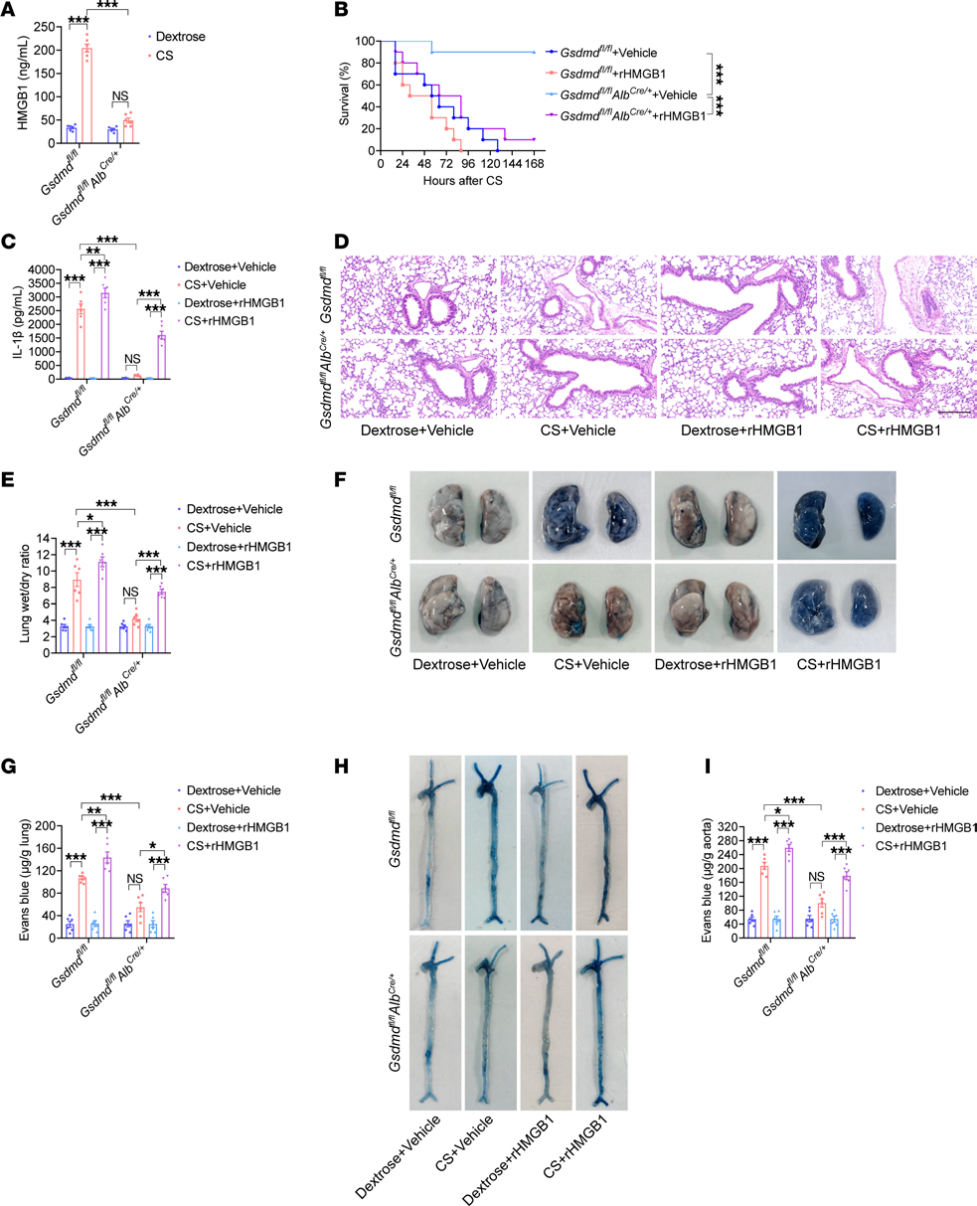
Hepatocyte *Gsdmd* deficiency inhibits HMGB1 release and vascular injury in sepsis. *Gsdmd^fl/fl^Alb^Cre/+^* and *Gsdmd^fl/fl^* mice were intraperitoneally injected with CS (2.5 mg/g) or 5% dextrose. (**A**) The plasma HMGB1 level was determined after treatment with CS or 5% dextrose for 16 hours. *n* = 6 per group. The data are shown as the means ± SEMs. Two-way ANOVA with Bonferroni’s post hoc correction was performed. *Gsdmd^fl/fl^* *Alb^Cre/+^* and *Gsdmd^fl/fl^* mice were intraperitoneally injected with CS (2.5 mg/g). The vehicle control or rHMGB1 protein was subsequently administered intravenously at 2, 16, 28, and 40 hours after CS injection. (**B**) Mouse survival was assessed and is shown as a Kaplan-Meier plot. *n* = 10 per group. A log-rank (Mantel-Cox) test was used. *Gsdmd^fl/fl^* *Alb^Cre/+^* and *Gsdmd^fl/fl^* mice were intraperitoneally injected with CS (2.5 mg/g) or 5% dextrose. Then, vehicle control or 5 μg of rHMGB1 protein was administered intravenously at 2 and 16 hours, and the blood, aortas, and lungs were excised from the mice and analyzed. (**C**) The plasma IL-1β level was determined. *n* = 6 per group. (**D**) HE staining of the lung sections. The scale bar represents 200 μm. (**E**) The lung wet/dry ratio was quantitatively analyzed. *n* = 6 per group. (**F**) Lung microvascular permeability was determined. (**G**) The lung Evans blue dye content was quantified. *n* = 6 per group. (**H**) Aortic permeability was determined. (**I**) The aortic Evans blue dye content was quantitatively analyzed. *n* = 6 per group. The data are expressed as the means ± SEMs. The data were analyzed by 2-way ANOVA with Bonferroni’s post hoc correction. All the data shown are representative of a minimum of 3 independent experiments. *P < 0.05, **P < 0.01, ***P < 0.001.

Compared with that in *Gsdmd^fl/fl^* mice, the CS-induced plasma HMGB1 level was significantly lower in *Gsdmd^fl/fl^* *Alb^Cre/+^* mice, and no significant difference in the circulating HMGB1 concentration was detected between *Gsdmd^fl/fl^* *Alb^Cre/+^* mice administered CS or 5% dextrose (Figure 7A). Compared with those in *Gsdmd^fl/fl^* mice, improved survival and significantly decreased plasma IL-1β concentrations were observed in *Gsdmd^fl/fl^* *Alb^Cre/+^* mice with sepsis, and these effects were reversed by rHMGB1 protein injection (Figure 7, B and C). Compared with those in *Gsdmd^fl/fl^* mice, lung edema, lung microvascular permeability, and aortic permeability were obviously inhibited in *Gsdmd^fl/fl^* *Alb^Cre/+^* mice with sepsis, and these effects were reversed by the injection of the rHMGB1 protein (Figure 7, D–I). Collectively, these results suggest that hepatocytic GSDMD is responsible for the release of HMGB1, ultimately resulting in systemic vascular injury and lethality in sepsis.

### Targeting endothelial GSDMD protected against systemic vascular injury and lethality in endotoxemia and sepsis.

**Figure 8:**
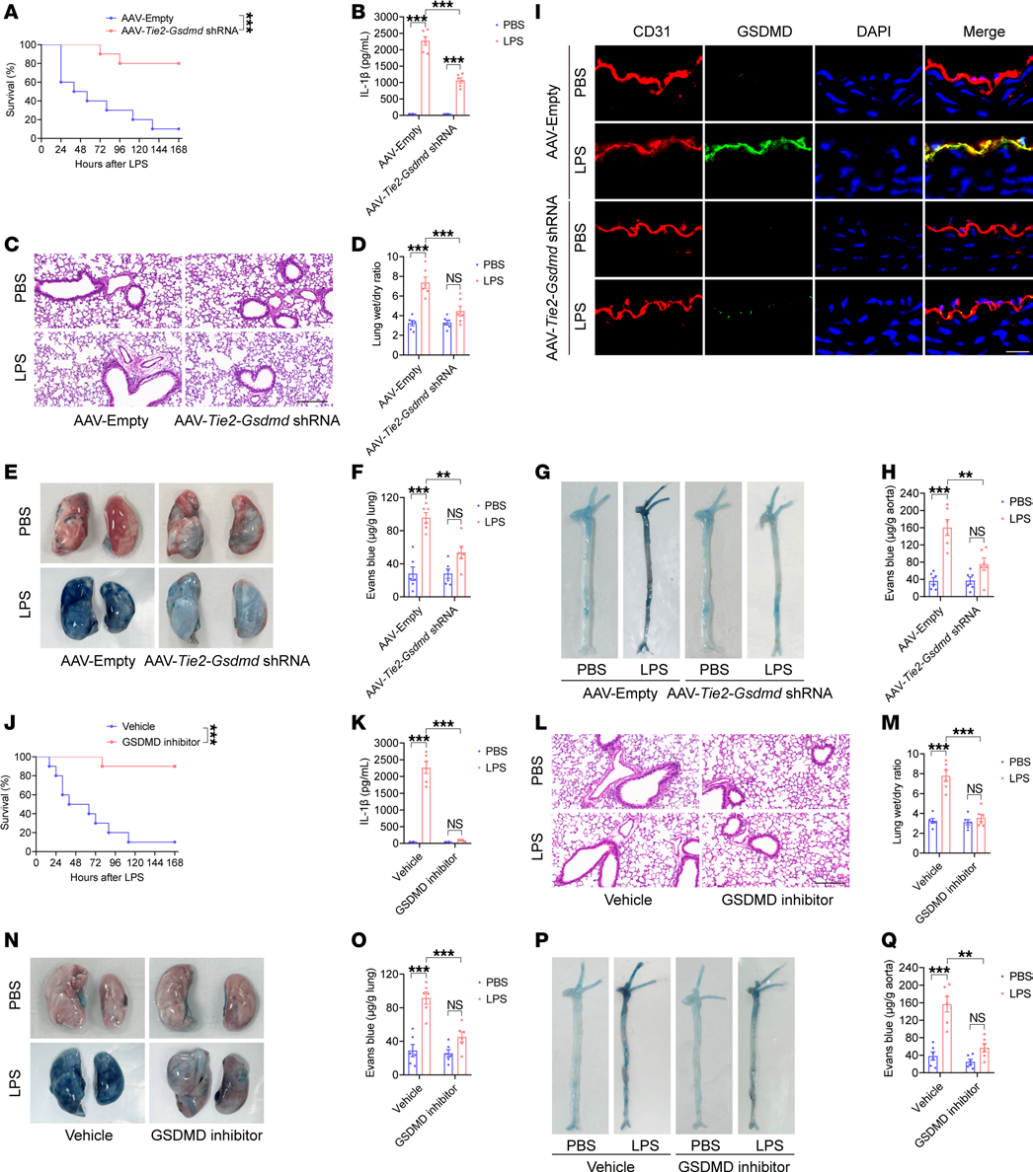
Inhibiting endothelial GSDMD activation improves endothelial barrier integrity and survival in endotoxemia. Five-week-old WT mice were injected with a null AAV9 vector or an endothelial conditional *Gsdmd* shRNA-knockdown AAV9 vector via the tail vein. After 6 weeks, these mice were intraperitoneally injected with LPS (17.5 mg/kg) or PBS. In addition, WT mice were intraperitoneally injected with LPS (17.5 mg/kg). The 200 μg GSDMD inhibitor or vehicle control was subsequently administered intraperitoneally at 2, 12, 24, and 36 hours after LPS. Blood, aortas, and lungs were obtained from the mice after treatment with LPS or PBS for 16 hours and were subsequently assessed. (**A** and **J**) A Kaplan-Meier survival plot of mice. *n* = 10 per group. Survival data were analyzed by a log-rank (Mantel-Cox) test. (**B** and **K**) The plasma IL-1β concentration was determined. *n* = 6 per group. (**C** and **L**) Representative HE-stained images of the lung sections are presented. The scale bar represents 200 μm. (**D** and **M**) The ratio of the wet lung weight to the dry lung weight was determined. *n* = 6 per group. (**E** and **N**) Lung microvascular permeability was detected. (**F** and **O**) The lung Evans blue dye content was quantified. *n* = 6 per group. (**G** and **P**) Aortic permeability was determined. (**H** and **Q**) The aortic Evans blue dye content was quantitatively analyzed. *n* = 6 per group. The data are expressed as the means ± SEMs. The data were analyzed by 2-way ANOVA with Bonferroni’s post hoc correction. (**I**) Representative immunofluorescence images of CD31 (red), GSDMD (green), and DAPI (blue) staining in aortas. The scale bar represents 20 μm. All the data shown are representative of a minimum of 3 independent experiments. **P < 0.01, ***P < 0.001.

GSDMD has emerged as a promising therapeutic target for the treatment of LPS-triggered endotoxemia (44, 45). Five-week-old mice were injected with an endothelium-specific *Gsdmd* shRNA-knockdown AAV9 vector via the tail vein and then treated with LPS after 6 weeks. Compared with the null AAV9 vector, the endothelium-specific *Gsdmd* shRNA-knockdown AAV9 vector significantly improved the survival of the mice from 10% to 80% and decreased the release of IL-1β during endotoxemia (Figure 8, A and B). Compared with those in the null AAV9 vector–treated mice, LPS-induced pulmonary edema and pulmonary microvascular permeability were evidently alleviated in the endothelium-conditioned *Gsdmd* shRNA-knockdown AAV9 vector–treated mice (Figure 8, C–F). The endothelium-conditioned *Gsdmd* shRNA–mediated knockdown of the AAV9 vector also reduced aortic permeability and endothelial GSDMD levels in LPS-treated mice (Figure 8, G–I).

The mouse GSDMD recognition motif for inflammatory caspases has been reported, which indicates that the GSDMD cleavage site peptide LLSD directly binds to caspase-11 (29, 46). We designed and synthesized a GSDMD activation inhibitor, benzyloxycarbonyl-Leu-Leu-Ser-Asp-fluoromethyl ketone, which targets LLSD from GL Biochem Co., Ltd. and was previously demonstrated to successfully suppress pyroptosis (47). This GSDMD activation inhibitor was administered intraperitoneally at a dose of 200 μg at 2, 12, 24, and 36 hours after LPS injection. Compared with vehicle injection, the use of a GSDMD activation inhibitor significantly reduced the plasma HMGB1 concentration in endotoxemic mice, which was comparable to the effect of *Gsdmd* siRNA injection (Supplemental Figure 9A). LPS-induced death and circulating IL-1β levels were significantly lower in the GSDMD activation inhibitor group than in the vehicle group (Figure 8, J and K). Compared with the vehicle, the GSDMD activation inhibitor significantly reduced LPS-stimulated lung edema, lung microvascular permeability, and aortic permeability (Figure 8, L–Q).

We also identified the protective role of a GSDMD activation inhibitor during sepsis. Compared with the vehicle, the GSDMD activation inhibitor obviously reduced the mortality rate and plasma HMGB1 and IL-1β concentrations in the mice with sepsis (Supplemental Figure 9B and Supplemental Figure 10, A and B). Compared with the vehicle, the GSDMD activation inhibitor significantly inhibited pulmonary edema, pulmonary microvascular permeability, and aortic permeability in mice with sepsis (Supplemental Figure 10, C–F). Similar improvements were observed in sepsis model mice after the use of an endothelially conditioned *Gsdmd* shRNA-knockdown AAV9 vector (Supplemental Figure 10, G–L). Therefore, inhibiting endothelial GSDMD expression and activation decreased vascular injury and improved survival in mice with endotoxemia or sepsis.

## Discussion

The endothelium is recognized as a fully fledged organ that covers an area of nearly 1,000 m^2^ (5, 48). The amount of evidence supporting the role of endothelial cells in both physiological and pathological responses to sepsis is continuously growing (6). Endothelial cells make up 50% of lung cells and are initially exposed to bacteria in the blood, making the lungs the most vulnerable organs to septic damage (4, 49). ALI, including its most severe manifestation, acute respiratory distress syndrome, is a major complication and a leading cause of sepsis-related death in clinical practice (50, 51). Pathologically, ALI is characterized by damage to the microvascular endothelium and alveolar epithelium and alveolar capillary leakage and fluid accumulation in the alveolar and interstitial space, leading to inflammatory cell infiltration and edema formation (50, 52). Endotoxemia has ongoing utility in preclinical research, specifically in examining the acute response associated with the initial stages of sepsis (53). Hence, a better understanding of the regulatory mechanisms of endothelial damage in endotoxemia and endotoxemia-induced septic lethality is urgently needed. In this study, we demonstrated that endothelial GSDMD, not myeloid cell–derived GSDMD, was responsible for endothelial injury–mediated ALI and lethality in endotoxemia and sepsis. In contrast with previous studies that focused on lung microvascular changes (25, 54), our results revealed increased aortic permeability in endotoxemia and sepsis, which was prevented by endothelial *Gsdmd* deletion. The results of this study suggested that endothelial GSDMD-mediated endothelial pyroptosis causes systemic vascular injury, which may trigger systemic hypoperfusion and organ dysfunction, ultimately leading to death in endotoxemia or sepsis.

In this study, endothelial *Gsdmd* deficiency protected mice against LPS-induced ALI and death but did not reduce the IL-1β concentration to the baseline level. Inconsistent with previous research (54), our results suggest that the IL-1β level is not a determining factor in endotoxemia-induced ALI or lethality. A cytokine storm is a life-threatening systemic inflammatory syndrome involving elevated levels of circulating cytokines and immune cell hyperactivation that can be triggered by LPS and sepsis (55, 56). Cytokine storms can lead to multiorgan dysfunction and even multiorgan failure and lethality if inadequately treated (55). Myeloid cells, including monocytes, mature macrophages, and granulocytes, are the primary sources of IL-1 family members and tumor necrosis factor–α (TNF-α), which are closely associated with cytokine storm disorders and GSDMD-mediated pyroptosis (9, 55, 57–59). In response to acute infections, the production and mobilization of monocyte and neutrophil populations from the bone marrow increase, and these cells are recruited to sites of inflammation to produce and release IL-1 and TNF-α (60). Although some studies have reported that neutralizing antibodies against both TNF-α and IL-1β improved survival to approximately 60% after intraperitoneal injection of LPS in mice (61, 62), neutralization of IL-1β via an IL-1 receptor antagonist did not protect against LPS-induced organ dysfunction (63). Randomized controlled clinical trials have also indicated the failure of TNF-α or IL-1 blockade to improve outcomes in sepsis patients (64–66). We found that conditional endothelial deletion of *Gsdmd* completely protected against mortality and obviously alleviated ALI in mice with endotoxemia, while myeloid cell *Gsdmd* deficiency did not improve ALI or survival. Therefore, endothelial GSDMD activation–mediated endothelial pyroptosis is most likely the decisive cause of endotoxemia-induced death, whereas the cytokine storm driven mainly by the myeloid cell line may aggravate the disease.

Huebener et al. (67) reported that hepatocyte *Hmgb1* deficiency reduced circulating HMGB1 levels in LPS-treated mice but had no influence on LPS-induced lethal shock. These results were different from those published by Wang et al. (22) and Denget al. (21), in which 70% and 90% of the LPS-treated mice survived following treatment with an HMGB1-neutralizing antibody and hepatocyte-specific *Hmgb1* deletion, respectively. Our data revealed that survival improved from 10% to 90% in hepatocyte-conditioned *Hmgb1*-deficient mice with endotoxemia, which was also inconsistent with the findings of Huebener et al. (67). The release of HMGB1 is regulated by GSDMD activation (68). The direct effects of hepatocyte *Gsdmd* deletion on circulating HMGB1 levels and survival in LPS-treated mice have not been explored. In our study, the increase in liver GSDMD activation preceded the increase in circulating HMGB1 levels during endotoxemia, and hepatocyte-specific *Gsdmd* deficiency decreased the plasma HMGB1 concentration and improved the survival of mice from 10% to 100%. Therefore, the inhibition of hepatocyte GSDMD had a greater protective effect on endotoxemia than did hepatocyte-specific *Hmgb1* knockout, and other factors that are released may depend on hepatocyte GSDMD activation and have a lethal effect on endotoxemia.

Our results indicated that both hepatocyte-specific *Gsdmd* deletion and endothelial *Gsdmd* deletion prevented LPS-induced death. Although in vitro experiments have shown that HMGB1 derived from hepatocytes delivers LPS into the cytosol of lung endothelial cells to trigger caspase-11–dependent pyroptosis (21), the regulatory mechanism between hepatocyte GSDMD and vascular endothelial GSDMD in vivo needs further clarification. We demonstrated that hepatocyte GSDMD activation occurred earlier than vascular endothelial GSDMD activation in LPS-treated mice. In endotoxemia, we demonstrated that hepatocyte GSDMD was responsible for regulating the release of HMGB1. Additionally, we found that hepatocyte *Gsdmd* deletion inhibited LPS-induced vascular endothelial GSDMD levels and systemic vascular injury and that these effects were reversed by rHMGB1 protein intervention. Therefore, the results of these in vivo experiments verified that hepatocyte GSDMD mediated HMGB1 release and subsequently regulated endothelial GSDMD-mediated vascular injury in endotoxemia.

Currently, researchers in the field agree that LPS injection may serve as a model for endotoxic shock but not for sepsis (69). LPS is a single component of complex pathogen-associated molecular patterns released by Gram-negative organisms (42). LPS injection neglects the host-pathogen interactions of Gram-positive organisms and polymicrobial sepsis (42). Numerous clinical trials of antiinflammatory strategies for the treatment of sepsis might be referred to as “graveyards” for pharmaceutical companies, since almost none of these strategies has resulted in obviously improved survival of patients (69). Cecal contents contain not only live microbes but also particulate matter that assists with bacterial colonization of the peritoneum (42). In an untreated animal, bacterial colonies can be recovered transiently from the blood and persist in the peritoneum and visceral organs (42). We used CS to establish a peritoneal sepsis model and further investigated the role of endothelial GSDMD in sepsis. Consistent with our findings in LPS shock, hepatocyte GSDMD-mediated HMGB1 contributed to endothelial GSDMD-mediated systemic vascular damage in sepsis, and endothelial *Gsdmd* deficiency prevented sepsis-induced lethality.

These data suggest that endothelial GSDMD may be an attractive target for treating endotoxemia and sepsis. We used an endothelial cell–specific *Gsdmd* shRNA-knockdown AAV9 vector to inhibit endothelial GSDMD levels, thereby protecting mice with endotoxemia and sepsis from ALI and mortality. We designed and synthesized a GSDMD activation inhibitor based on the possible conserved inflammatory caspase cleavage site in mouse GSDMD (29). We previously demonstrated the inhibitory effect of this inhibitor on GSDMD activation by determining LPS-induced lactate dehydrogenase release and propidium iodide staining in vitro (47). Here, we used a GSDMD inhibitor to prevent endothelial injury, systemic vascular injury, and lethality in mice with endotoxemia and sepsis successfully. Therefore, this research validates endothelial GSDMD as a viable pharmaceutical target and provides a basis for the development of future therapeutics for endotoxemia and endotoxemia-induced septic lethality.

There are several limitations to this study. We found that endothelial *Gsdmd* deletion, rather than myeloid cell *Gsdmd* deletion, prevented vascular injury and death in sepsis. Consistently, the IL-1β concentration in endothelial *Gsdmd*-knockout mice was greater than that in myeloid cell *Gsdmd*-knockout mice. Therefore, the effects of IL-1β on sepsis remain to be further determined. In addition, it remains unknown whether the protective role of endothelial *Gsdmd* deletion is associated with endothelial pyroptosis-related cytokine storms in sepsis.

Although excessive or uncontrolled pyroptosis has a deleterious effect on the host, it has proven to have a game-changing therapeutic effect on pathogenic invasion when controlled (70). As a critical mechanism of host defense, GSDMD activation–mediated IL-18 release contributes to the killing and clearance of gastrointestinal pathogens in intestinal cells and immune cells, which drives anti-rotavirus immunity and protects mice against rotavirus infection (71). GSDMD-mediated pyroptosis promotes the Th1 immune response by controlling the release of IL-18, which plays a vital role in clearing the parasite (72). Upon GSDMD activation, GSDMD-N and other cytosolic contents are released from pyroptotic cells and reduce the number of intracellular and extracellular bacteria by causing host cell death or a direct antibacterial effect (70, 73). *Gsdmd* deficiency led to severe abscess formation, extensive skin damage, bacterial spread, and cellular inflammation in a mouse model of *S*. *aureus* skin infection (74). The activation of GSDMD-dependent pyroptosis and IL-18 secretion has been shown to improve antitumor immunity by maintaining healthy gut microbiota (71, 75, 76). However, further experiments are needed to determine the role of GSDMD in host defense in the future (77).

## Methods

### Sex as a biological variable.

Our study examined male and female animals, and similar findings are reported for both sexes.

### Animals.

C57BL/6J (WT) mice (No. 219) were purchased from Charles River Laboratories Co., Ltd. (Beijing, China). *Gsdmd^–/–^* mice and *Gsdmd^fl/fl^* mice generated via the CRISPR/Cas9 system were purchased from GemPharmatech Co., Ltd. (Nanjing, China) (78). *Hmgb1^fl/fl^* mice were gifted by Tadatsugu Taniguchi from the University of Tokyo, Tokyo, Japan (20). *Alb^Cre/+^* mice (Stock No. 003574), *Tie2^Cre/+^* mice (Stock No. 008863), and *Lyz2^Cre/+^* mice (Stock No. 004781) were purchased from The Jackson Laboratory (Bar Harbor, Maine, USA) (79–81). *Alb^Cre/+^*, *Tie2^Cre/+^*, and *Lyz2^Cre/+^* mice were crossed with *Gsdmd^fl/fl^* mice to generate *Gsdmd^fl/fl^* *Alb^Cre/+^* mice, *Gsdmd^fl/fl^* *Tie2^Cre/+^* mice, and *Gsdmd^fl/fl^* *Lyz2^Cre/+^* mice, respectively, and their *Cre*-negative littermates (*Gsdmd^fl/fl^* mice). *Alb^Cre/+^* mice were crossed with *Hmgb1^fl/fl^* mice to generate *Hmgb1^fl/fl^* *Alb^Cre/+^* mice and their *Cre*-negative littermates (*Hmgb1^fl/fl^* mice). WT mice, *Gsdmd^–/–^* mice, *Gsdmd^fl/fl^* *Alb^Cre/+^* mice, *Gsdmd^fl/fl^* *Tie2^Cre/+^* mice, *Gsdmd^fl/fl^* *Lyz2^Cre/+^* mice, *Gsdmd^fl/fl^* mice, *Hmgb1^fl/fl^* *Alb^Cre/+^* mice, and *Hmgb1^fl/fl^* mice were used for in vivo experiments. The mice were housed under a 12-hour light/12-hour dark cycle in a temperature-controlled specific pathogen–free environment with ad libitum access to a regular chow diet and water. The genotypes were confirmed via PCR as previously described (82). For identification of the *Gsdmd*-floxed allele, the primers used were as follows: forward primer, TCTGTTCCCTCCAGCCCTACTTG; reverse primer, CAGCAACCACAGCACTACGTTC. The WT allele corresponded to a band of 223 bp, and the floxed allele yielded a product of 321 bp. The forward primer CGATGGAACGTAGTGCTGTG and reverse primer TCCTTCCCAACCTGCTGTTG were used for genotyping the *Gsdmd^–/–^* mice. The WT allele yielded a band of 550 bp, and the conventional knockout allele yielded a band of 423 bp. The forward primer AGCGATGGATTTCCGTCTCTGG and the reverse primer AGCTTGCATGATCTCCGGTATTGAA were used to examine *Alb-Cre* transgenic mice, *Tie2-Cre* transgenic mice, and *Lyz2-Cre* transgenic mice, which resulted in a band of 272 bp, whereas *Cre*-negative mice presented no band. For identification of the *Hmgb1*-floxed allele, the primers used were as follows: forward primer, TGTCATGCCACCCTGAGCAGTT; reverse primer, TGTGCTCCTCCCGGCAAGTT. The WT allele corresponded to a 172 bp band, and the floxed allele yielded a 280 bp product.

### Endotoxic shock model.

For LPS-induced endotoxemia, 10- to 12-week-old WT mice, *Gsdmd^–/–^* mice, *Gsdmd^fl/fl^* *Alb^Cre/+^* mice, *Gsdmd^fl/fl^* *Tie2^Cre/+^* mice, *Gsdmd^fl/fl^* *Lyz2^Cre/+^* mice, *Gsdmd^fl/fl^* mice, *Hmgb1^fl/fl^* *Alb^Cre/+^* mice, and *Hmgb1^fl/fl^* mice were injected intraperitoneally with a lethal dose of LPS (17.5 mg/kg). LPS-induced endotoxemia was also established via intratracheal delivery. Briefly, WT mice between 10 and 12 weeks of age were anesthetized and subjected to intratracheal administration of LPS (17.5 mg/kg) in 50 μL of sterile PBS. The survival of the mice was observed and recorded every 8 hours.

### CS model of sepsis.

Sepsis was induced in the mice via the CS method described by Rincon et al. (42). Briefly, 10- to 12-week-old WT mice were euthanized, the skin was opened, and the cecum was excised. The cecal contents were suspended in 5% dextrose to produce a final concentration of 60 mg/mL CS. For sepsis, 10- to 12-week-old WT mice, *Gsdmd^–/–^* mice, *Gsdmd^fl/fl^* *Alb^Cre/+^* mice, *Gsdmd^fl/fl^* *Tie2^Cre/+^* mice, *Gsdmd^fl/fl^* *Lyz2^Cre/+^* mice, and *Gsdmd^fl/fl^* mice were injected intraperitoneally with a dose of CS (2.5 mg/g). The survival of the mice was observed and recorded every 8 hours.

### Vascular permeability measurements.

An Evans blue–albumin extravasation assay was performed to assess endothelial permeability (25, 83, 84). Briefly, anesthetized mice were injected with 50 μL of 7% Evans blue dye (No. E2129, Merck) via retro-orbital injection. After 10 minutes, the intravascular Evans blue dye was removed via PBS perfusion (20 mL) through the left ventricle. Mouse aortas (from the aortic arch to the iliac arteries) and lungs were harvested, air-dried, weighed, homogenized, and extracted in 0.5 mL and 1 mL formamide (No. 47671, Merck), respectively, for 24 hours at 60°C. The quantity of Evans blue dye in the aorta and lung homogenate supernatants was determined spectrophotometrically at an absorbance of 620 nm. The Evans blue dye content is expressed as micrograms per gram (μg/g) of aorta or lung.

### Determination of the lung wet/dry weight ratio.

The lungs were excised from the mice, and wet weights were immediately measured. After the samples were dried at 60°C for 48 hours, the dry weights were measured. The lung wet/dry weight ratio was then calculated (85–87).

### AAV9 vector injection.

A *Rage* shRNA-knockdown AAV9 vector, a *Tlr4* shRNA-knockdown AAV9 vector, a *Gsdmd* shRNA-knockdown AAV9 vector, and a null AAV9 vector as a negative control harbored a *Tie2* promoter, which contributed to the specific knockdown of target genes in endothelial cells. A *Rage* shRNA-mediated knockdown AAV9 vector and a null AAV9 vector harbored an *Sp-c* promoter, which induced specific knockdown of target genes in type II alveolar epithelial cells. All the recombinant AAV9 vectors were constructed by Genomeditech Co., Ltd. (Shanghai, China). Five-week-old WT mice were injected with 100 μL of virus containing 1 × 10^12^ vector genomes via the tail vein.

### siRNA injection.

The *Gsdmd*-siRNA and negative control used for tail vein injection were provided by BiOligo Biotechnology Co., Ltd. (Shanghai, China). Each mouse received a freshly prepared mixture (10 nmol of siRNA dissolved in 200 μL of saline) on days 2, 4, and 6 before endotoxemia or sepsis model construction.

### Histopathology.

The mice were euthanized under deep anesthesia 16 hours after treatment with LPS or CS. Aortas (from the aortic arch to the iliac arteries), and lungs were collected and fixed in 4% paraformaldehyde (No. G1101, Servicebio) for 24 hours. The tissues were then dehydrated through a graded ethanol series, embedded in paraffin wax, and finally sectioned into 3 μm sections. The sections were dewaxed and stained with hematoxylin and eosin (No. G1076, Servicebio), dehydrated in ethanol and *n*-butanol, and cleared in xylene before being mounted with neutral balsam. Images were captured using a Leica microscope.

Dehydrated paraffin sections were pretreated via heat-mediated antigen retrieval with antigen repair buffer (No. B0035, POWERFUL). The slides were incubated with 5% BSA (No. A8010, Solarbio) at room temperature for 30 minutes and stained with a rabbit anti-GSDMD antibody (1:200, No. NBP2-33422, Novus) and a goat anti-platelet endothelial cell adhesion molecule (CD31) antibody (1:200, No. AF3628, R&D Systems, Bio-Techne) overnight at 4°C. The sections were washed 3 times with PBS (No. G0002-2 L, Servicebio), followed by a 1-hour incubation at room temperature with an Alexa Fluor 488–conjugated donkey anti-rabbit IgG (H+L) cross-adsorbed secondary antibody (1:400, No. A-21206, Invitrogen) and an Alexa Fluor 555–conjugated donkey anti-goat IgG (H+L) cross-adsorbed secondary antibody (1:400, No. A-21432, Invitrogen). Nuclei were stained with antifade mounting medium containing DAPI (No. B0025, POWERFUL) at room temperature for 10 minutes. Images were acquired via a Nikon microscope.

All histopathological assessments were performed by researchers who were masked to the experimental groups.

### Western blotting.

Mouse aortas (from the aortic arch to the iliac arteries) and livers were surgically removed and lysed as previously described (82). Proteins (20–40 μg) were separated on 10% or 12.5% sodium dodecyl sulfate-polyacrylamide gels (No. PG113, EpiZyme) and transferred onto polyvinylidene difluoride membranes (No. ISEQ00010, MilliporeSigma). The membranes were blocked with protein-free rapid blocking buffer (No. PS108P, EpiZyme) for 10 minutes at room temperature and incubated overnight at 4°C with primary antibodies, including mouse anti-GSDMD (1:500, No. sc-393656, Santa Cruz Biotechnology) and β-actin (1:20,000, No. BS6007MH, Bioworld) antibodies. The membranes were incubated with Peroxidase AffiniPure goat anti-mouse IgG (H+L) (1:5,000, No. 115-035-003, Jackson ImmunoResearch) secondary antibody for 2 hours at room temperature. Bands were visualized via enhanced chemiluminescence (No. RPN2235, Cytiva). Band intensity was quantified via Quantity One software (Bio-Rad), and the data were normalized against those of β-actin.

### ELISA.

Blood was collected following cardiac puncture and centrifuged at 1,006*g* at 4°C for 15 minutes. The levels of IL-1β and HMGB1 in mouse plasma were determined via a commercially available IL-1β ELISA Kit (No. EK201B, MULTISCIENCES) and an HMGB1 ELISA Kit (No. ARG81310 and No. ARG81351, Arigo) according to the manufacturer’s instructions.

### Statistics.

Statistical analysis was performed via GraphPad Prism software, version 8.0. The quantitative data are shown as the means ± SEMs. A log-rank (Mantel-Cox) test was used to compare survival curves. When the data were normally distributed and the variances between groups were equal, data were further analyzed with an unpaired 2-tailed Student’s *t* test for comparisons of 2 groups or with 1-way ANOVA followed by Bonferroni’s post hoc correction for the comparison of multiple groups. Analyses of the effects of different treatments on mice with different phenotypes were performed via 2-way ANOVA with Bonferroni’s post hoc correction. A *P* value less than 0.05 was considered significant.

### Study approval.

All the animal studies were approved by the Institutional Animal Care and Use Committee of the Shanghai Research Center for Model Organisms, Shanghai, China. All experimental procedures involving animals were performed in accordance with the NIH *Guide for the Care and Use of Laboratory Animals* (NIH Publication No. 85–23, revised 1996, National Academies Press).

### Data availability.

The values for all the data points in the graphs are reported in the Supporting Data Values file.

## Author contributions

ES, HJ, and ML conceptualized the study. ES, XS, and LW analyzed the data. ES, XS, LW, JX, XC, SX, and ML performed experiments. ES and XS wrote the original draft of the manuscript. ES, XS, LW, and JX reviewed and edited of the manuscript. HJ and ML supervised the study. We assigned the authorship according to the number of tasks undertaken by the author.

## Supplementary Material

Supplemental data

Unedited blot and gel images

Supporting data values

## Version 1 02/10/2025

Electronic publication
