# Supplementary material for: Endothelial GSDMD underlies LPS-induced systemic vascular injury and lethality
Source: JCI Insight. 2025 Feb 10;10(3):e182398. doi: 10.1172/jci.insight.182398 (PMC11948583; doi:10.1172/jci.insight.182398)
Supplement: Supplemental data [file jciinsight-10-182398-s009.pdf]

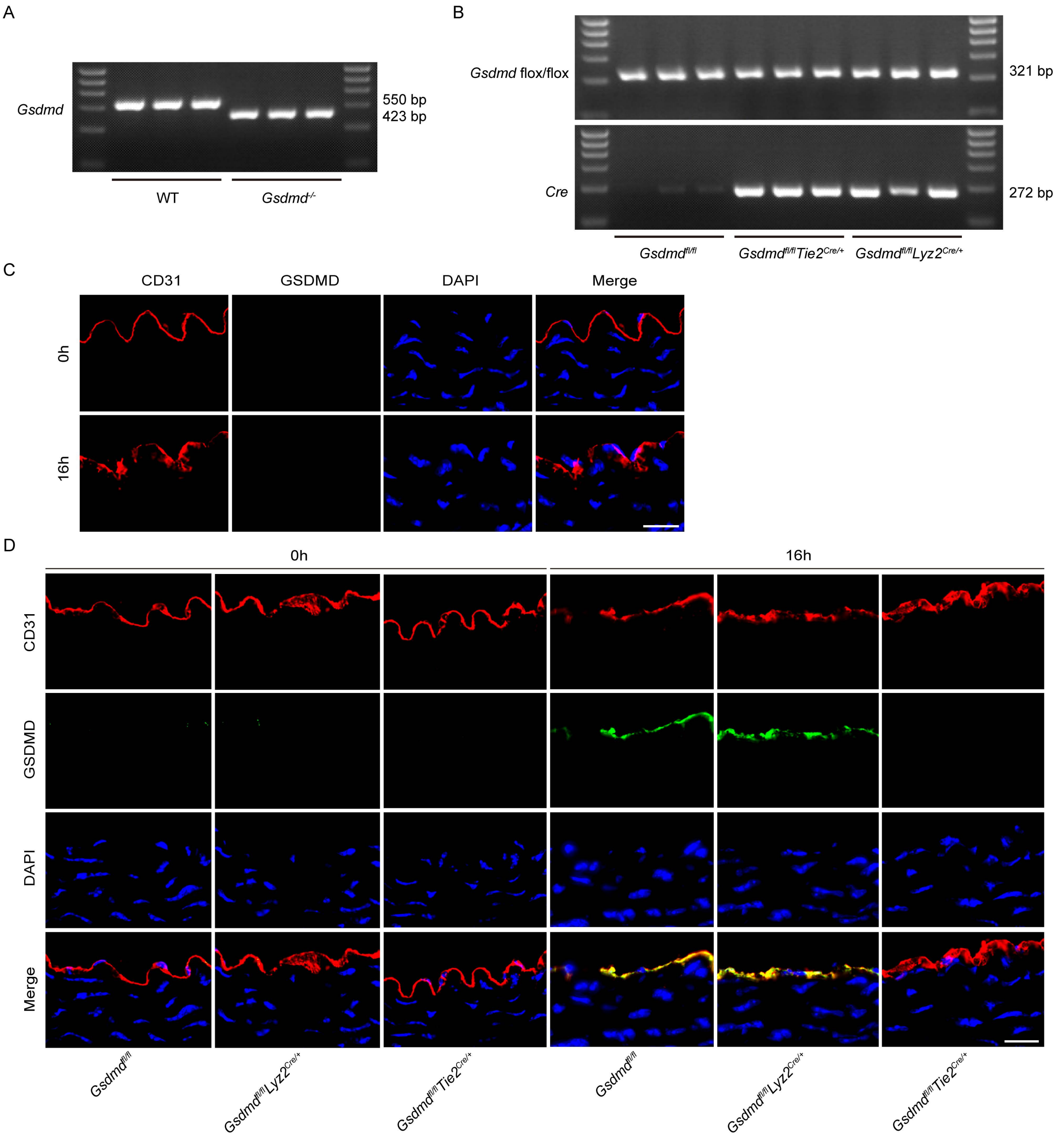
A
Gsdmd
550 bp
423 bp
WT
Gsdmd-/-
B
Gsdmd flox/flox
321 bp
Cre
272 bp
Gsdmdfl/fl
Gsdmdfl/flTie2Cre/+
Gsdmdfl/flLyz2Cre/+
C
CD31
GSDMD
DAPI
Merge
0h
16h
D
0h
16h
CD31
GSDMD
DAPI
Merge
Gsdmdfl/fl
Gsdmdfl/flLyz2Cre/+
Gsdmdfl/flTie2Cre/+
Gsdmdfl/fl
Gsdmdfl/flLyz2Cre/+
Gsdmdfl/flTie2Cre/+

Supplemental Figure 1. The level of endothelial GSDMD in global *Gsdmd* deletion mice and tissue-conditioned *Gsdmd* knockout mice with endotoxemia. Genotyping of **(A)** wild-type (WT) mice and global *Gsdmd* knockout ($Gsdmd^{-/-}$) mice and **(B)** endothelial *Gsdmd*-deficient ($Gsdmd^{fl/fl}Tie2^{Cre/+}$) mice, myeloid cell *Gsdmd*-deficient ($Gsdmd^{fl/fl}Lyz2^{Cre/+}$) mice and their *Cre*-negative littermates ($Gsdmd^{fl/fl}$ mice). $Gsdmd^{-/-}$ mice, $Gsdmd^{fl/fl}Tie2^{Cre/+}$ mice, $Gsdmd^{fl/fl}Lyz2^{Cre/+}$ mice and $Gsdmd^{fl/fl}$ mice were intraperitoneally injected with a lethal dose of LPS (17.5 mg/kg). Representative aortic immunofluorescence images of CD31 (red), GSDMD (green) and DAPI (blue) at 0 h and 16 h after exposure to LPS in **(C)** $Gsdmd^{-/-}$ mice, **(D)** $Gsdmd^{fl/fl}Tie2^{Cre/+}$ mice, $Gsdmd^{fl/fl}Lyz2^{Cre/+}$ mice and $Gsdmd^{fl/fl}$ mice. The scale bar represents 20 μm.

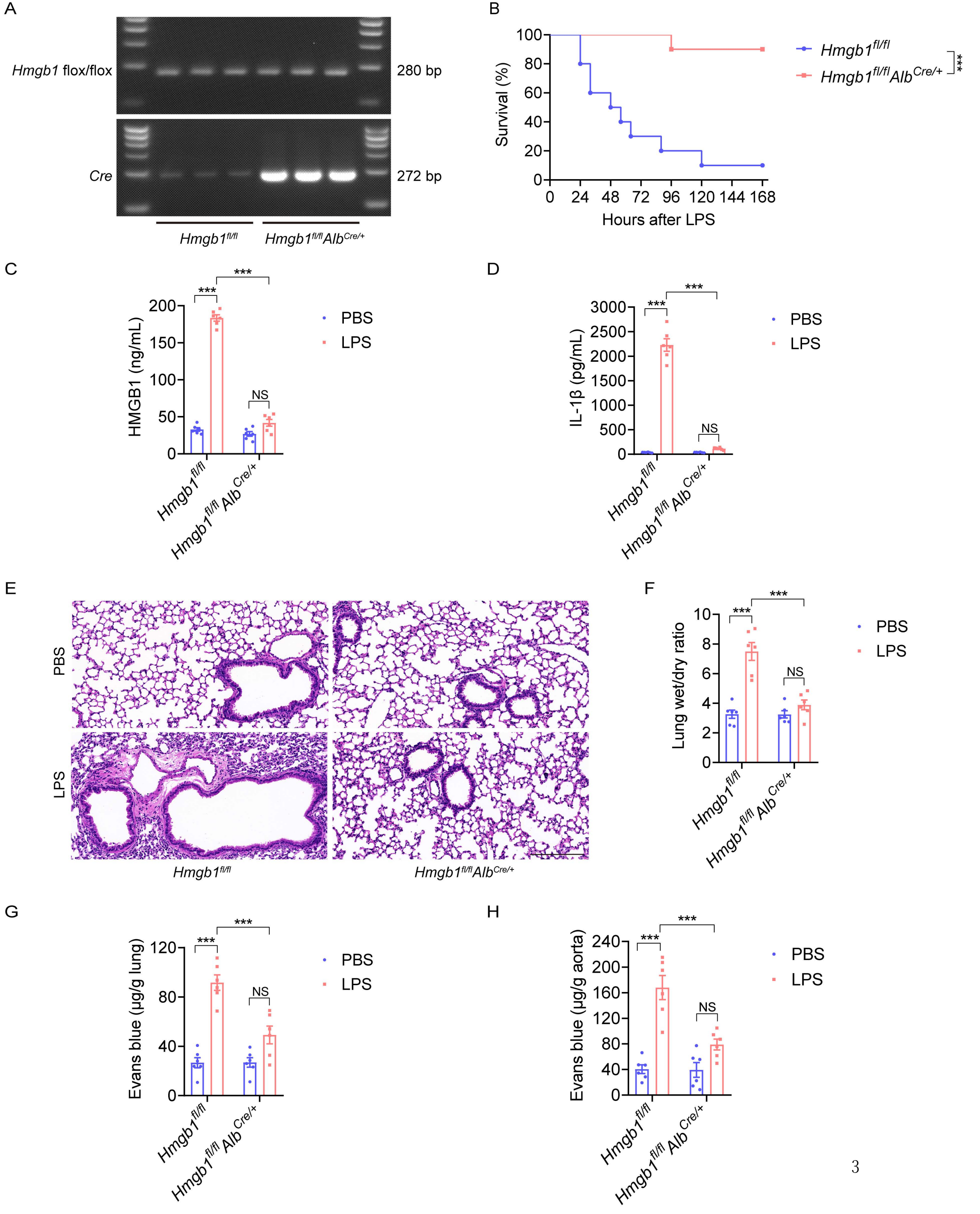
A
Hmgb1 flox/flox
280 bp
Cre
272 bp
Hmgb1fl/fl
Hmgb1fl/flAlbCre/+
B
Survival (%)
Hours after LPS
Hmgb1fl/fl
Hmgb1fl/flAlbCre/+
***
C
HMGB1 (ng/mL)
PBS
LPS
***
NS
D
IL-1β (pg/mL)
PBS
LPS
***
NS
E
PBS
LPS
Hmgb1fl/fl
Hmgb1fl/flAlbCre/+
F
Lung wet/dry ratio
PBS
LPS
***
NS
G
Evans blue (μg/g lung)
PBS
LPS
***
NS
H
Evans blue (μg/g aorta)
PBS
LPS
***
NS

Supplemental Figure 2. Hepatocyte *Hmgb1* deletion reduces circulating HMGB1 levels and vascular injury in endotoxemia. (A) Genotyping of hepatocellular *Hmgb1*-deficient ($Hmgb1^{fl/fl}Alb^{Cre/+}$) mice and their *Cre*-negative littermates ($Hmgb1^{fl/fl}$ mice). $Hmgb1^{fl/fl}Alb^{Cre/+}$ mice and $Hmgb1^{fl/fl}$ mice were intraperitoneally injected with LPS (17.5 mg/kg). **(B)** Mouse survival was assessed on the indicated days and is shown as a Kaplan–Meier plot. n=10 per group. Survival data were analyzed by a log-rank (Mantel–Cox) test. Blood, aortas and lungs were obtained from $Hmgb1^{fl/fl}Alb^{Cre/+}$ mice and $Hmgb1^{fl/fl}$ mice after treatment with LPS (17.5 mg/kg) or PBS for 16 h and were analyzed. The plasma **(C)** HMGB1 and **(D)** IL-1β levels were determined in the indicated groups. n=6 per group. The data are shown as the means ± SEMs. Two-way ANOVA with Bonferroni post hoc correction was performed. **(E)** Representative HE-stained images of the lung sections are presented. The scale bar represents 200 µm. **(F)** The lung wet/dry ratio was quantitatively analyzed. n=6 per group. Lung microvascular permeability and aortic permeability were detected via an Evans blue–albumin extravasation assay. **(G)** The amount of extracted pulmonary Evans blue dye in the formamide extracts was quantified by measuring the dye at 620 nm. n=6 per group. **(H)** The aortic Evans blue dye content was quantitatively analyzed. n=6 per group. The data are shown as the means ± SEMs. Two-way ANOVA with Bonferroni post hoc correction was used. All the data shown are representative of a minimum of 3 independent experiments. ***$P < 0.001$.

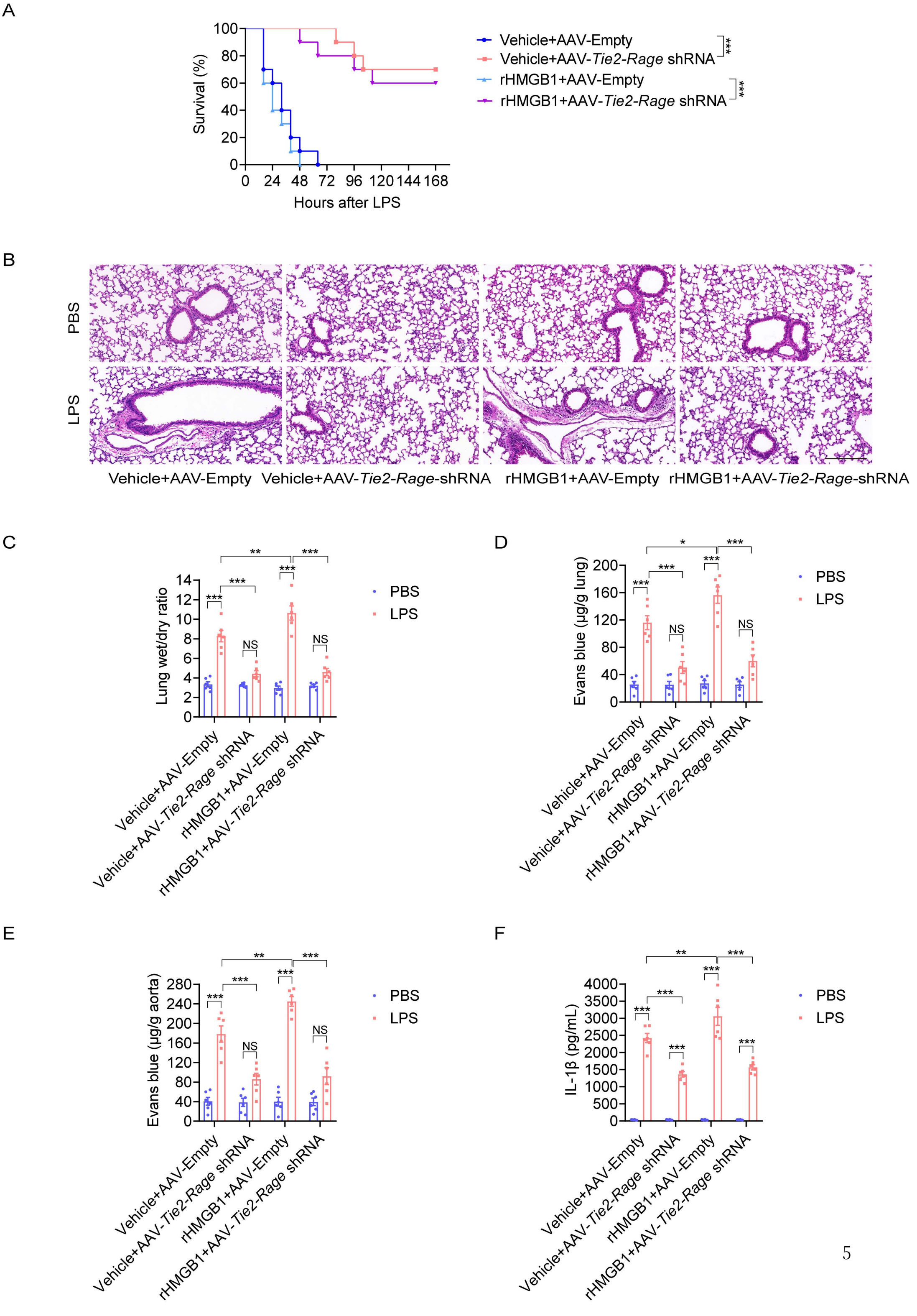
A
Survival (%)
100
80
60
40
20
0
0 24 48 72 96 120 144 168
Hours after LPS
Vehicle+AAV-Empty
Vehicle+AAV-Tie2-Rage shRNA
rHMGB1+AAV-Empty
rHMGB1+AAV-Tie2-Rage shRNA
***
***
B
PBS
LPS
Vehicle+AAV-Empty
Vehicle+AAV-Tie2-Rage-shRNA
rHMGB1+AAV-Empty
rHMGB1+AAV-Tie2-Rage-shRNA
C
Lung wet/dry ratio
PBS
LPS
Vehicle+AAV-Empty
Vehicle+AAV-Tie2-Rage shRNA
rHMGB1+AAV-Empty
rHMGB1+AAV-Tie2-Rage shRNA
D
Evans blue (μg/g lung)
PBS
LPS
E
Evans blue (μg/g aorta)
PBS
LPS
F
IL-1β (pg/mL)
PBS
LPS
NS
*
**
***

Supplemental Figure 3. The HMGB1/RAGE axis regulated vascular injury and death in mice intratracheally instilled with LPS. Five-week-old WT mice were injected with a null AAV9 vector or an endothelial conditional *Rage* shRNA-knockdown AAV9 vector via the tail vein. After 6 weeks, these mice were intratracheally instilled with LPS (17.5 mg/kg). Subsequently, vehicle or Recombinant HMGB1 (rHMGB1) protein was administered intravenously at a dose of 5 μg at 2, 16, 28, and 40 h after LPS injection. **(A)** Mouse survival was monitored on the indicated days and is presented as a Kaplan–Meier plot. n=10 per group. Survival data were compared via a log-rank (Mantel–Cox) test. Five-week-old WT mice were injected with a null AAV9 vector or an endothelial conditional *Rage* shRNA-knockdown AAV9 vector via the tail vein and were intratracheally instilled with LPS (17.5 mg/kg) or PBS after 6 weeks. Then, vehicle or rHMGB1 protein was administered intravenously at a dose of 5 μg at 2 and 16 h, and the blood, aortas and lungs were excised from the mice and analyzed. **(B)** HE staining of the lung sections is presented. The scale bar represents 200 μm. **(C)** The ratio of the wet lung weight to the dry lung weight was determined. n=6 per group. Lung microvascular permeability and aortic permeability were detected via an Evans blue–albumin extravasation assay. **(D)** The amount of extracted pulmonary Evans blue dye in the formamide extracts was quantified by measuring the dye at 620 nm. n=6 per group. **(E)** The aortic Evans blue dye content was quantitatively analyzed. n=6 per group. **(F)** The plasma IL-1β concentration was determined. n=6 per group. The data are expressed as the means ± SEMs. The data were analyzed by two-way ANOVA with Bonferroni post hoc correction. All the data shown are representative of a minimum of 3 independent experiments. $^{*}P < 0.05$, $^{**}P < 0.01$, $^{***}P < 0.001$.

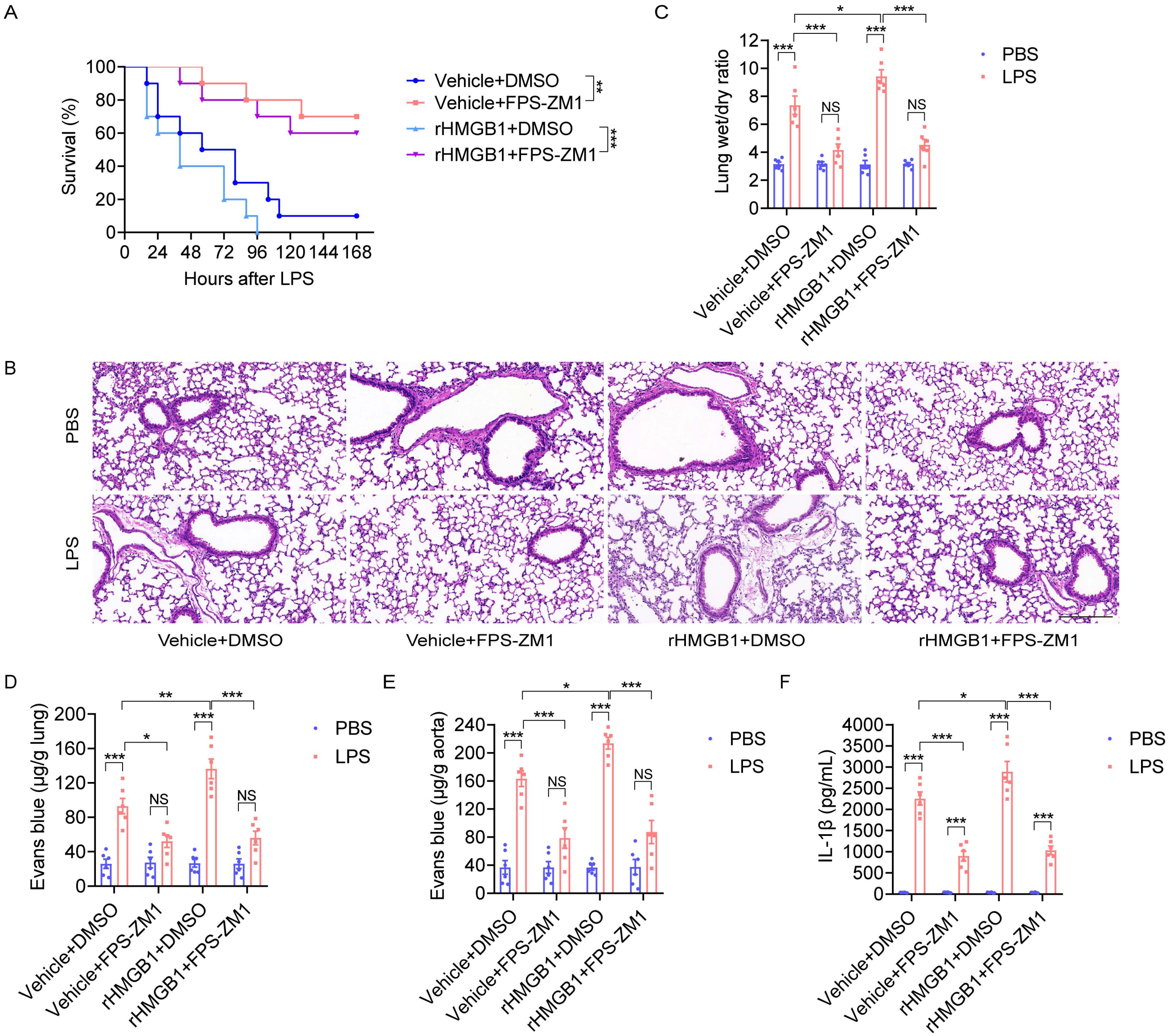
A
Survival (%)
Hours after LPS
Vehicle+DMSO
Vehicle+FPS-ZM1
rHMGB1+DMSO
rHMGB1+FPS-ZM1
**
***
B
PBS
LPS
Vehicle+DMSO
Vehicle+FPS-ZM1
rHMGB1+DMSO
rHMGB1+FPS-ZM1
C
Lung wet/dry ratio
PBS
LPS
NS
D
Evans blue (μg/g lung)
E
Evans blue (μg/g aorta)
F
IL-1β (pg/mL)

Supplemental Figure 4. HMGB1 binding to RAGE is responsible for systemic vascular injury and death in endotoxemia. WT mice were intraperitoneally injected with FPS-ZM1 (3 mg/kg) or dimethyl sulfoxide (DMSO) at 72, 48, 24, and 1 h before LPS (17.5 mg/kg) injection and at 24, 48, 72, 96, 120, 144 and 168 h after LPS injection. Subsequently, vehicle or rHMGB1 protein was administered intravenously at a dose of 5 μg at 2, 16, 28, and 40 h after LPS injection. **(A)** Mouse survival was monitored on the indicated days and is presented as a Kaplan–Meier plot. n=10 per group. Survival data were compared via a log-rank (Mantel–Cox) test. WT mice were intraperitoneally injected with FPS-ZM1 (3 mg/kg) or DMSO at 72, 48, 24, and 1 h before LPS (17.5 mg/kg) or PBS injection. Then, vehicle or rHMGB1 protein was administered intravenously at a dose of 5 μg at 2 and 16 h, and the blood, aortas and lungs were excised from the mice and analyzed. **(B)** HE staining of the lung sections is presented. The scale bar represents 200 μm. **(C)** The ratio of the wet lung weight to the dry lung weight was determined. n=6 per group. Lung microvascular permeability and aortic permeability were detected via an Evans blue–albumin extravasation assay. **(D)** The amount of extracted pulmonary Evans blue dye in the formamide extracts was quantified by measuring the dye at 620 nm. n=6 per group. **(E)** The aortic Evans blue dye content was quantitatively analyzed. n=6 per group. **(F)** The plasma IL-1β concentration was determined. n=6 per group. The data are expressed as the means ± SEMs. The data were analyzed by two-way ANOVA with Bonferroni post hoc correction. All the data shown are representative of a minimum of 3 independent experiments. *$P < 0.05$, **$P < 0.01$, ***$P < 0.001$.

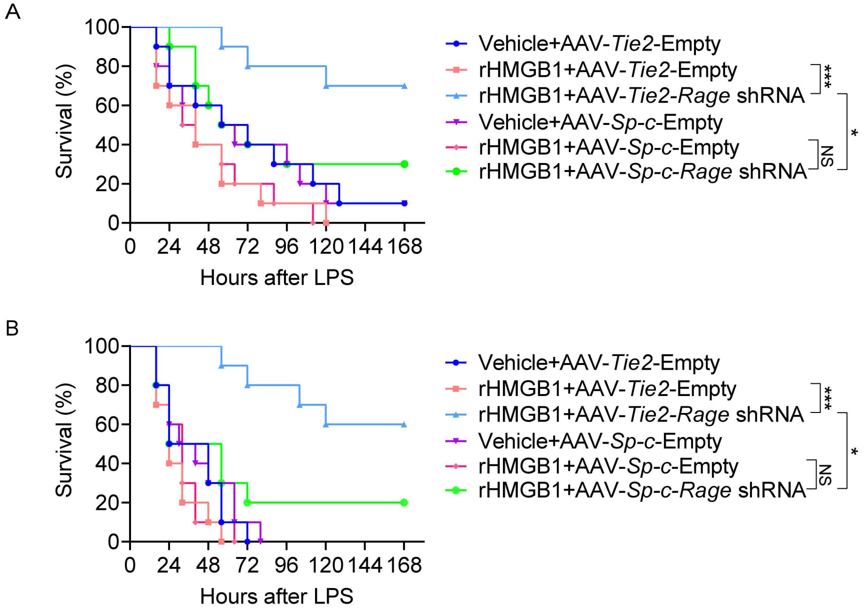

Supplemental Figure 5. The role of type II alveolar epithelial RAGE in endotoxemia. Five-week-old WT mice were injected with a null AAV9 vector, an endothelial conditional *Rage* shRNA-knockdown AAV9 vector or a type II alveolar epithelial conditional *Rage* shRNA-knockdown AAV9 vector via the tail vein. After 6 weeks, these mice were intraperitoneally injected with LPS (17.5 mg/kg) or intratracheally instilled with LPS (17.5 mg/kg). Subsequently, vehicle or rHMGB1 protein was administered intravenously at a dose of 5 μg at 2, 16, 28, and 40 h after LPS injection or instillation. The survival of the mice **(A)** that were intraperitoneally injected with LPS or **(B)** intratracheally instilled with LPS was monitored on the indicated days and is presented as a Kaplan–Meier plot. n=10 per group. Survival data were compared via a log-rank (Mantel–Cox) test. All the data shown are representative of a minimum of 3 independent experiments. *$P < 0.05$, ***$P < 0.001$.

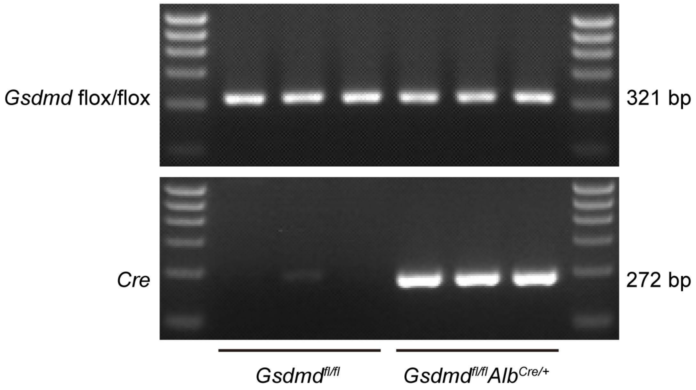

Supplemental Figure 6. Construction of hepatocyte-specific *Gsdmd*-deficient mice. Genotyping of hepatocellular *Gsdmd*-deficient (*Gsdmd*$^{fl/fl}$*Alb*$^{Cre/+}$) mice and their *Cre*-negative littermates (*Gsdmd*$^{fl/fl}$ mice).

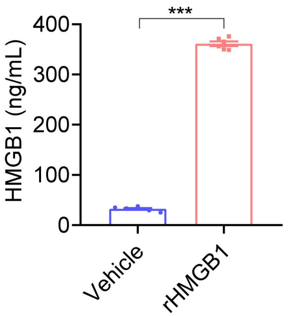

Supplemental Figure 7. The plasma HMGB1 level after rHMGB1 protein injection. *Gsdmd*$^{fl/fl}$*Alb*$^{Cre/+}$ mice were intraperitoneally injected with LPS (17.5 mg/kg). Subsequently, vehicle or rHMGB1 protein was administered intravenously at a dose of 5 μg at 2 and 16 h after LPS injection. The HMGB1 concentration in the plasma was subsequently measured. n=6 per group. The data are expressed as the means ± SEMs. An unpaired 2-tailed Student *t* test was performed. All the data shown are representative of a minimum of 3 independent experiments. ***$P < 0.001$.

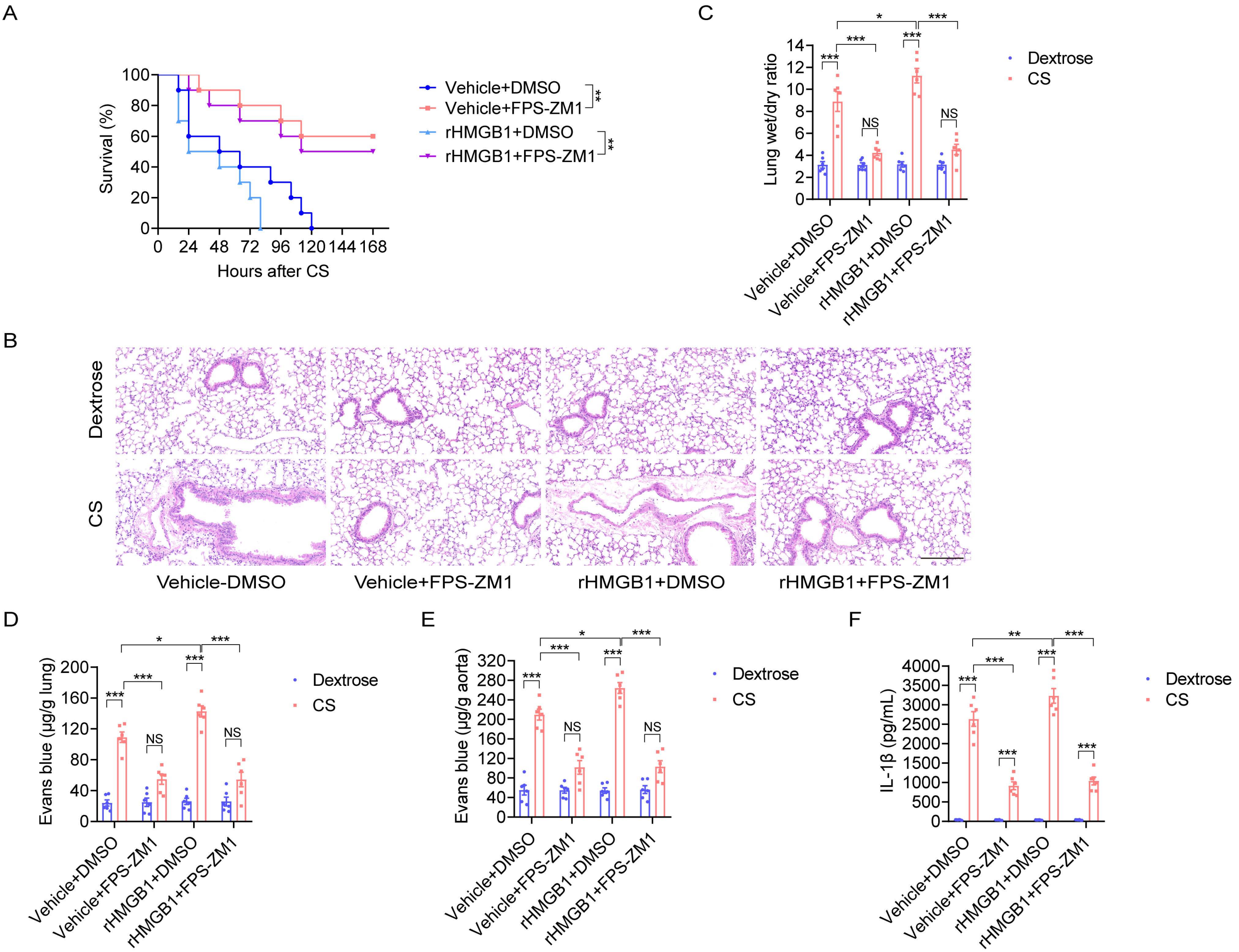
A
Survival (%)
Hours after CS
Vehicle+DMSO
Vehicle+FPS-ZM1
rHMGB1+DMSO
rHMGB1+FPS-ZM1
**
**
C
Lung wet/dry ratio
Dextrose
CS
***
*
NS
B
Dextrose
CS
Vehicle-DMSO
Vehicle+FPS-ZM1
rHMGB1+DMSO
rHMGB1+FPS-ZM1
D
Evans blue (μg/g lung)
E
Evans blue (μg/g aorta)
F
IL-1β (pg/mL)

Supplemental Figure 8. HMGB1 binding to RAGE causes systemic vascular injury and death in sepsis. WT mice were intraperitoneally injected with FPS-ZM1 (3 mg/kg) or DMSO at 72, 48, 24, and 1 h before cecal slurry (CS) (2.5 mg/g) injection and at 24, 48, 72, 96, 120, 144 and 168 h after CS injection. Subsequently, vehicle or rHMGB1 protein was administered intravenously at a dose of 5 μg at 2, 16, 28, and 40 h after CS injection. **(A)** Mouse survival was monitored on the indicated days and is presented as a Kaplan–Meier plot. n=10 per group. Survival data were compared via a log-rank (Mantel–Cox) test. WT mice were intraperitoneally injected with FPS-ZM1 (3 mg/kg) or DMSO at 72, 48, 24, and 1 h before CS (2.5 mg/g) or 5% dextrose injection. Then, vehicle or rHMGB1 protein was administered intravenously at a dose of 5 μg at 2 and 16 h, and the blood, aortas and lungs were excised from the mice and analyzed. **(B)** HE staining of the lung sections is presented. The scale bar represents 200 μm. **(C)** The ratio of the wet lung weight to the dry lung weight was determined. n=6 per group. Lung microvascular permeability and aortic permeability were detected via an Evans blue–albumin extravasation assay. **(D)** The amount of extracted pulmonary Evans blue dye in the formamide extracts was quantified by measuring the dye at 620 nm. n=6 per group. **(E)** The aortic Evans blue dye content was quantitatively analyzed. n=6 per group. **(F)** The plasma IL-1β concentration was determined. n=6 per group. The data are expressed as the means ± SEMs. The data were analyzed by two-way ANOVA with Bonferroni post hoc correction. All the data shown are representative of a minimum of 3 independent experiments. *$P < 0.05$, **$P < 0.01$, ***$P < 0.001$.

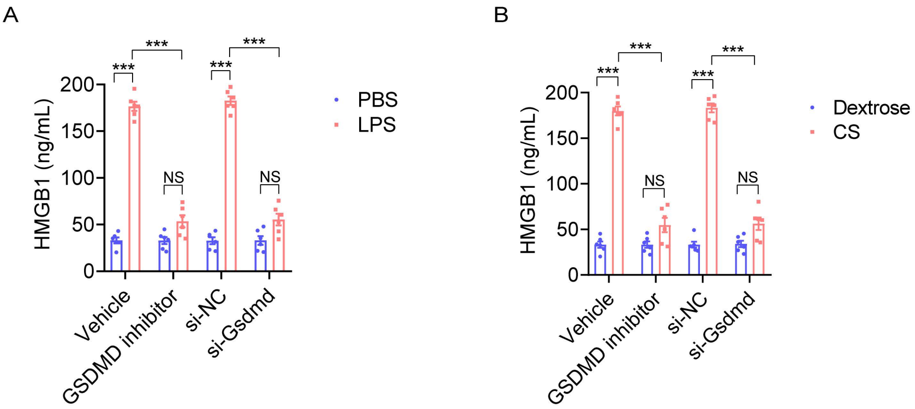

Supplemental Figure 9. Effects of a GSDMD activation inhibitor on plasma HMGB1 levels in endotoxemia and sepsis. WT mice were intraperitoneally injected with LPS (17.5 mg/kg) or PBS. Then, the vehicle control or GSDMD inhibitor was administered intraperitoneally at a dose of 5 μg at 2 and 12 h. In addition, WT mice were injected with *Gsdmd*-siRNA (si-*Gsdmd*) (10 nmol) or negative control (si-NC) (10 nmol) on days 2, 4, and 6 via the tail vein before LPS (17.5 mg/kg) or PBS injection. Blood was excised from the mice and analyzed after treatment with LPS or PBS for 16 h. **(A)** The plasma HMGB1 concentration was determined. n=6 per group. WT mice were intraperitoneally injected with CS (2.5 mg/g) or 5% dextrose. Then, the vehicle control or GSDMD inhibitor was administered intraperitoneally at a dose of 5 μg at 2 and 12 h. In addition, WT mice were injected with *Gsdmd*-siRNA (si-*Gsdmd*) (10 nmol) or negative control (si-NC) (10 nmol) on days 2, 4, and 6 via the tail vein before CS (2.5 mg/g) or 5% dextrose injection. The blood was excised from the mice and analyzed after treatment with CS or 5% dextrose for 16 h. **(B)** The plasma HMGB1 concentration was determined. n=6 per group. The data are shown as the means ± SEMs. Two-way ANOVA with Bonferroni post hoc correction was used. All the data shown are representative of a minimum of 3 independent experiments. ***$P < 0.001$.

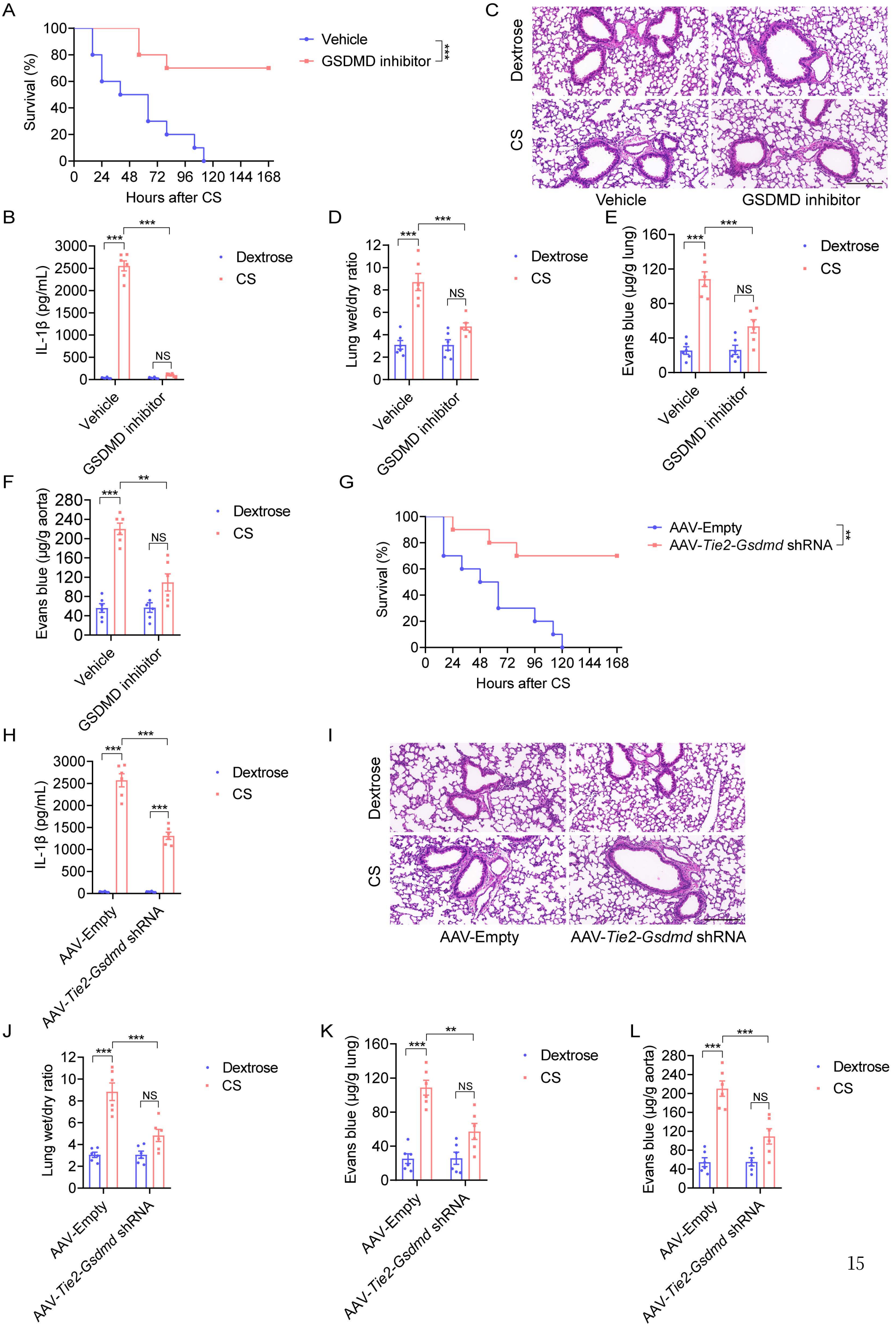

A
B
C
D
E
F
G
H
I
J
K
L
Vehicle
GSDMD inhibitor
Survival (%)
Hours after CS
Dextrose
CS
IL-1β (pg/mL)
Lung wet/dry ratio
Evans blue (μg/g lung)
Evans blue (μg/g aorta)
AAV-Empty
AAV-Tie2-Gsdmd shRNA
NS
***
**

Supplemental Figure 10. The GSDMD activation inhibitor alleviated systemic vascular injury and death in sepsis. WT mice were intraperitoneally injected with CS (2.5 mg/g). The vehicle control or GSDMD inhibitor was subsequently administered intraperitoneally at a dose of 200 μg at 2, 12, 24, and 36 h after CS. **(A)** Mouse survival was monitored on the indicated days and is presented as a Kaplan–Meier plot. n=10 per group. A log-rank (Mantel–Cox) test was used to compare survival curves. WT mice were intraperitoneally injected with CS (2.5 mg/g) or 5% dextrose. Then, the vehicle control or GSDMD inhibitor was administered intraperitoneally at a dose of 5 μg at 2 and 12 h. The blood, aortas and lungs were excised from the mice and analyzed after treatment with CS or 5% dextrose for 16 h. **(B)** The plasma IL-1β concentration was determined. n=6 per group. **(C)** HE staining of the lung sections. The scale bar represents 200 μm. **(D)** The lung wet/dry ratio was quantitatively analyzed. n=6 per group. Lung microvascular permeability and aortic permeability were detected via an Evans blue–albumin extravasation assay. **(E)** The amount of extracted pulmonary Evans blue dye in the formamide extracts was quantified by measuring the dye at 620 nm. n=6 per group. **(F)** The aortic Evans blue dye content was quantitatively analyzed. n=6 per group. The data are shown as the means ± SEMs. Two-way ANOVA with Bonferroni post hoc correction was used. Five-week-old WT mice were injected with a null AAV9 vector or an endothelial-conditioned *Gsdmd* shRNA-knockdown AAV9 vector via the tail vein. After 6 weeks, these mice were intraperitoneally injected with CS (2.5 mg/g). **(G)** A Kaplan–Meier survival plot of mice on the indicated days is presented. n=10 per group. Survival data were analyzed by a log-rank (Mantel–Cox) test. Five-week-old WT mice were injected with a null AAV9 vector or an endothelial conditional *Gsdmd* shRNA-knockdown AAV9 vector via the tail vein and were intraperitoneally injected with CS (2.5 mg/g) or 5% dextrose after 6 weeks. Blood, aortas and lungs were obtained from the mice after treatment

with CS or 5% dextrose for 16 h and were subsequently assessed. **(H)** The plasma IL-1β concentration was determined. n=6 per group. **(I)** Representative HE-stained images of the lung sections are presented. The scale bar represents 200 μm. **(J)** The ratio of wet lung weight to dry lung weight was determined. n=6 per group. Lung microvascular permeability and aortic permeability were detected via an Evans blue–albumin extravasation assay. **(K)** The amount of extracted pulmonary Evans blue dye in the formamide extracts was quantified by measuring the dye at 620 nm. n=6 per group. **(L)** The aortic Evans blue dye content was quantitatively analyzed. n=6 per group. The data are expressed as the means ± SEMs. The data were analyzed by two-way ANOVA with Bonferroni post hoc correction. All the data shown are representative of a minimum of 3 independent experiments. $^{**}P < 0.01$, $^{***}P < 0.001$.
