# Supplementary figures and images for: Endothelial GSDMD underlies LPS-induced systemic vascular injury and lethality
Source: JCI Insight. 2025 Feb 10;10(3):e182398. doi: 10.1172/jci.insight.182398 (PMC11948583; doi:10.1172/jci.insight.182398)

**Full unedited gel for Figure 1I**

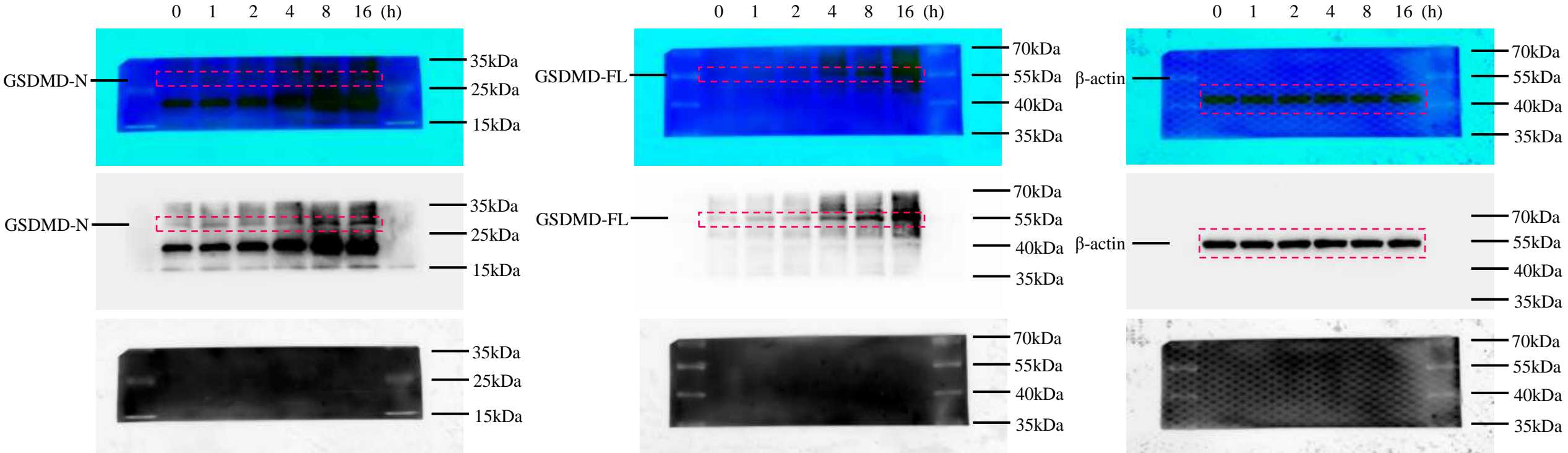

Full unedited gel for Figure 3A

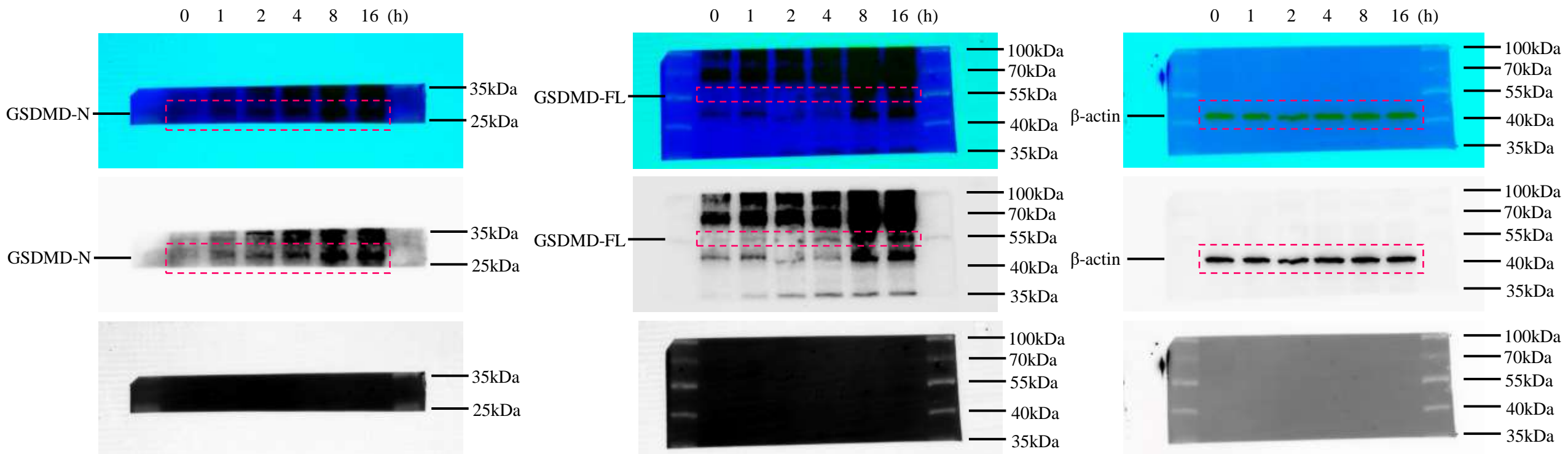

Supplement: Unedited blot and gel images [file jciinsight-10-182398-s010.pdf]
